# REST regulation of gene networks in adult neural stem cells

**DOI:** 10.1038/ncomms13360

**Published:** 2016-11-07

**Authors:** Shradha Mukherjee, Rebecca Brulet, Ling Zhang, Jenny Hsieh

**Affiliations:** 1 Department of Molecular Biology and Hamon Center for Regenerative Science and Medicine, University of Texas Southwestern Medical Center, Dallas, Texas 75390, USA

## Abstract

Adult hippocampal neural stem cells generate newborn neurons throughout life due to their ability to self-renew and exist as quiescent neural progenitors (QNPs) before differentiating into transit-amplifying progenitors (TAPs) and newborn neurons. The mechanisms that control adult neural stem cell self-renewal are still largely unknown. Conditional knockout of REST (repressor element 1-silencing transcription factor) results in precocious activation of QNPs and reduced neurogenesis over time. To gain insight into the molecular mechanisms by which REST regulates adult neural stem cells, we perform chromatin immunoprecipitation sequencing and RNA-sequencing to identify direct REST target genes. We find REST regulates both QNPs and TAPs, and importantly, ribosome biogenesis, cell cycle and neuronal genes in the process. Furthermore, overexpression of individual REST target ribosome biogenesis or cell cycle genes is sufficient to induce activation of QNPs. Our data define novel REST targets to maintain the quiescent neural stem cell state.

## Abstract (web summary)

The transcription factor REST plays a crucial role in maintaining the adult neural stem cell pool. To better understand how REST maintains quiescence in neural progenitors, the authors use ChIP-seq and RNA-seq and find that REST regulates represses ribosome biogenesis, cell cycle and neuronal genes.

## Graphical abstract

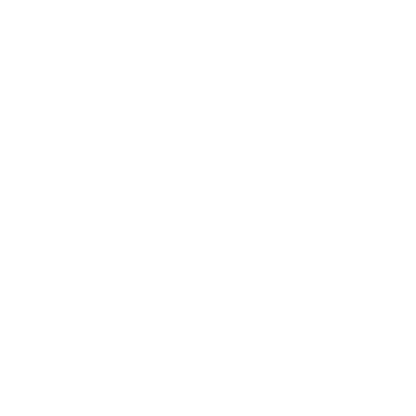

Quiescence is a cellular process to maintain long-lived self-renewing stem cells in a niche for continuous tissue replenishment^1^,^2^. An ideal niche to understand cellular quiescence is the subgranular zone of the hippocampal dentate gyrus^3^,^4^,^5^,^6^. Here slow-dividing quiescent neural progenitors (QNPs also known as type 1 or radial glial-like cells) undergo self-renewal to generate either proliferating ‘activated' QNPs or fast-dividing, transient-amplifying progenitors (TAPs also known as type 2 or non-radial cells) before differentiating into granule neurons in a process referred to as adult neurogenesis^7^,^8^,^9^. In response to external stimuli, such as physical exercise or seizure activity, each step in the process of neurogenesis is tightly regulated to yield functionally mature neurons with the potential to impact memory, depression and epilepsy^10^,^11^,^12^.

To understand the biology of QNPs and harness their therapeutic potential, it is important to identify the mechanisms that control quiescence and the transition to the proliferative state. Clonal analysis has shown that QNPs are multipotent and can generate neurons and astrocytes, and self-renew through both asymmetric and symmetric divisions^3^. While it is appreciated that QNPs integrate extrinsic and intrinsic signals to either maintain their quiescent state or become activated to divide and differentiate, the detailed mechanisms for these processes are still unknown.

Among the signalling pathways that govern QNP self-renewal, BMP signalling through BMPR-1A (ref. 13) and Notch1 signalling are essential for maintaining quiescence^14^,^15^, while canonical Wnt signalling promotes activation of QNPs and transition to the proliferative state by loss of Dkk1 or Sfrp3 inhibition in QNPs^16^,^17^. Moreover, recent studies have highlighted the important interplay between transcriptional and epigenetic mechanisms to regulate QNP self-renewal^18^. For example, the proneural transcription factor Ascl1 and the orphan nuclear receptor tailless promotes the proliferation of QNPs^19^,^20^,^21^,^22^ while the chromatin-modifying enzyme histone deacetylase 3 is required for the proliferation of TAPs^23^. Although there has been progress in identifying the gene regulatory networks in QNPs and TAPs, it is anticipated that additional transcriptional and epigenetic mechanisms work in concert to regulate self-renewal and proliferation^24^.

Previously, we showed that loss of repressor element 1-silencing transcription factor (REST), also known as neuron-restrictive silencer factor in adult hippocampal neural stem cells leads to precocious activation of QNPs and increased neurogenesis at an early time point^25^. When REST is conditionally removed in adult-born granule neurons, there is an overall reduction in neurogenesis over time. This early work raised the question of how REST regulates quiescence and the transition to proliferation. As REST is a negative regulator of gene expression, we hypothesized REST could potentially bind and regulate target genes involved in the maintenance of QNPs and the conversion of QNPs to TAPs.

Here we used genome-wide chromatin immunoprecipitation sequencing (ChIP-seq) and RNA-sequencing (RNA-seq) in adult neural stem cells to identify REST target genes in quiescent and proliferating conditions. Neuronal genes emerged as the most significant gene ontology (GO) category in unique QNP targets, unique TAP targets and targets common to both QNPs and TAPs. Furthermore, we identified non-neuronal REST target genes enriched in QNPs, such as regulators of ribosome biogenesis and cell cycle. To determine the role of REST quiescence effector genes, overexpression of individual REST target ribosome biogenesis or cell cycle genes was sufficient to promote activation of QNPs in cultured adult neural stem cells as well as in adult dentate gyrus. Overall, our work demonstrates that REST has a central role in maintaining both the quiescent and proliferation states of adult hippocampal neural stem cells by binding and regulating distinct target genes.

## Results

### REST is uniformly expressed in QNPs and TAPs *in vivo*

We previously reported that REST expression is present in QNPs, TAPs and mature granule neurons in the subgranular zone of the hippocampal dentate gyrus but is decreased in neuroblasts and immature neurons^25^. To further evaluate REST expression in the QNP population *in vivo*^3^,^26^, we performed immunohistochemical staining of REST using a ‘homemade' antibody (REST14) and a combination of cell type markers. We found significant co-localization of REST with GFAP+Sox2+ QNPs and GFAP+Ki67+ activated QNPs (Supplementary Fig. 1a,b,e,f). We found REST was expressed in the majority of Nestin+Ki67+ TAPs with a round soma and no radial process (Supplementary Fig. 1c,e,f). Consistent with previous work^25^, REST expression was downregulated in Nestin-GFP-Ki67+ neuroblasts and immature neurons expressing doublecortin (DCX) but was elevated in mature granule neurons expressing NeuN (Supplementary Fig. 1d,e,f). Altogether, these results demonstrate that REST is uniformly expressed in QNPs, activated QNPs and TAPs, and suggests it may have a key role in the maintenance of QNPs and the transition to TAPs *in vivo*.

### REST prevents QNP activation and transition to TAPs

**Figure 1:**
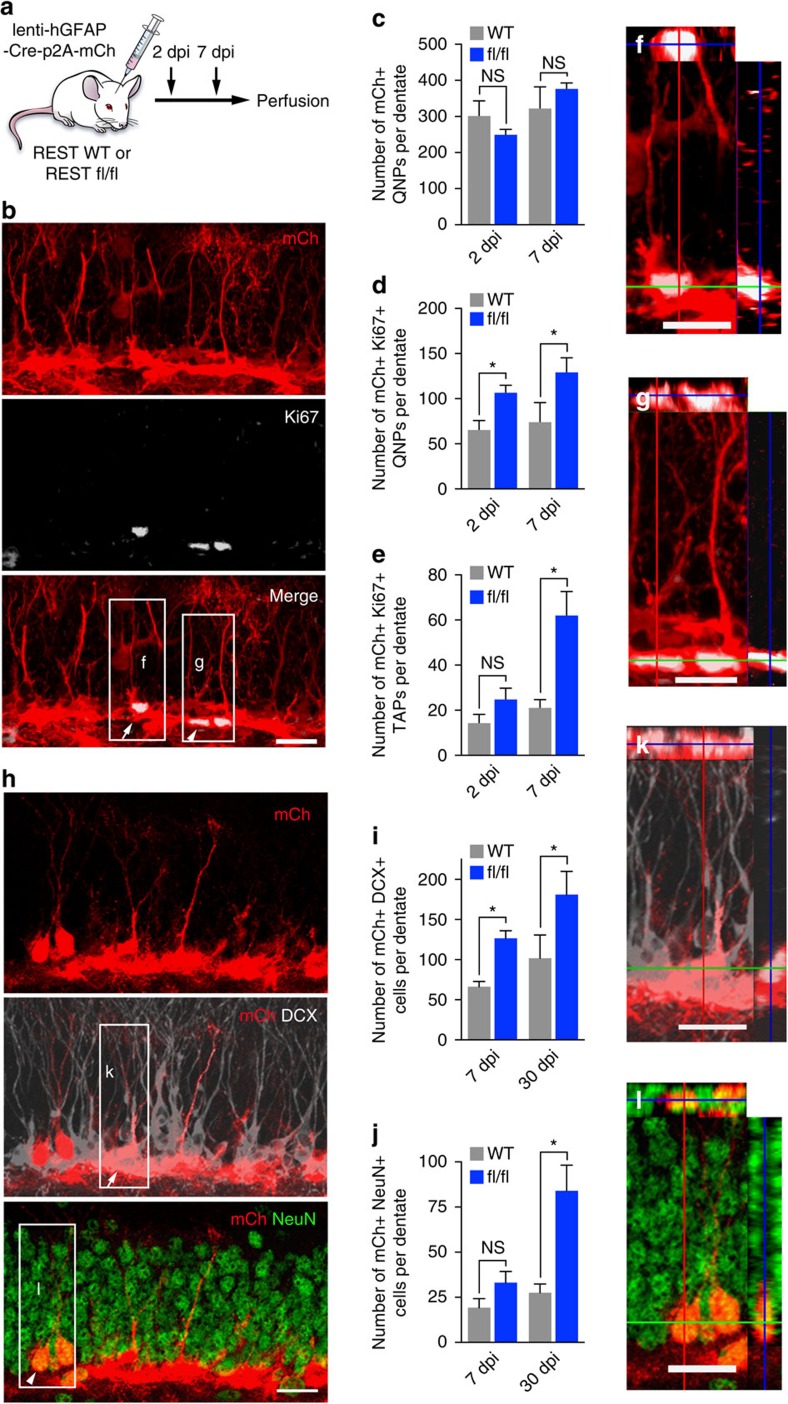
REST deletion in QNPs induces increased activation in adult hippocampus. (**a**) Lentivirus hGFAP-Cre-p2A-mCh injections were performed in hippocampal dentate gyrus of 6–8 week-old REST flox/flox (fl/fl) and WT mice. (**b**) Confocal images showing mCh+Ki67+ activated QNP (arrow) (inset f) and TAP (arrowhead) (inset g) cells at 2 dpi in REST fl/fl. (**c**) Quantification of the total number of mCh+ QNP cells per dentate gyrus. (**d**) The number of mCh+Ki67+ activated QNP cells at 2 dpi and 7 dpi. (**e**) The number of mCh+Ki67+ TAP cells at 2 dpi and 7 dpi. (**h**) Confocal images showing mCh+DCX+ immature neurons (arrow) (inset k) and mCh+NeuN+ mature neurons (arrowhead) (inset l) cells at 7 dpi in REST fl/fl. (**i**) The number of mCh+DCX+ immature neurons at 7 dpi and 30 dpi. (**j**) The number of mCh+NeuN+ mature neurons at 7 dpi and 30 dpi. For all quantifications, data are plotted as the mean±s.e.m. (*P≤0.05 and ns, not significant). Experiments were analysed for statistical significance using Kruskal–Wallis nonparametric test, which included Dunn's multiple comparison test and generated exact *P* values. Scale bar in **b**,**f**,**g**,**h**,**k** and **l**, 20 μm. At least 4–5 mice of each genotype were used. See also Supplementary Fig. 2.

We previously reported that conditional deletion of REST from Nestin-expressing stem/progenitors and their progeny using Nestin-CreER^T2^ transgenic mice crossed with REST flox/flox (fl/fl) mice led to precocious neuronal differentiation and depletion of the AH-NSC pool^25^. However, the Nestin-CreER^T2^ transgene is expressed in both QNP and TAP populations^27^, thus deleting REST in both quiescent and proliferating adult hippocampal neural stem cells. The question remained whether REST has a cell-autonomous role in QNPs. To address this question and specifically delete REST in QNPs *in vivo*, we delivered Cre-p2A-mCherry (mCh) lentivirus expressed under the control of the human GFAP (hGFAP) promoter (Fig. 1a). We performed immunohistochemical for Ki67 to determine the proliferation state of mCh+ QNPs or TAPs (Fig. 1b). We found that the dentate gyrus of REST fl/fl mice injected with Cre-encoding lentivirus contained a higher number of mCh+Ki67+ QNP cells compared with that of WT controls at both 2 and 7 dpi (Fig. 1b,d,f). To determine if precocious proliferation of QNPs after REST deletion led to an expansion of TAPs, we examined mCh+Ki67+ cells with TAP morphology located directly adjacent to mCh+Ki67+ QNPs. We hypothesized that as only a few Ki67+ TAPs expressed mCh at 2 dpi (Supplementary Fig. 1b,e,f), the majority of mCh+Ki67+ TAPs may result from the progeny of activated QNPs. We found that REST fl/fl mice injected with hGFAP-Cre-p2A-mCh lentivirus showed an increased number of mCh+Ki67+ TAP cells relative to control mice at 7 dpi, but not at 2 dpi, consistent with a time-dependent transition of TAPs from activated QNPs (Fig. 1b,e,g). These data are consistent with the cell-autonomous requirement of REST to maintain quiescence.

Differentiation of QNPs and TAPs to mature granule neurons takes around 28 days^28^. To address whether greater proliferation of QNPs and increased transition to TAPs after REST deletion leads to enhanced differentiation into neurons, we examined mCh+DCX+ immature neurons and mCh+NeuN+ mature neurons (Fig. 1h). We found that REST fl/fl mice injected with hGFAP-Cre-p2A-mCh lentivirus showed an increased number of mCh+DCX+ immature neurons relative to control mice at 7 dpi and 30 dpi (Fig. 1h,i,k). We also found an increased number of mCh+NeuN+ mature neurons in REST fl/fl relative to control mice at 30 dpi, but not at 7 dpi (Fig. 1h,j,l). These results support the conclusion that REST is required in QNPs to prevent the transition to TAPs and differentiated neurons.

### REST maintains TAP proliferation and prevents neurogenesis

**Figure 2:**
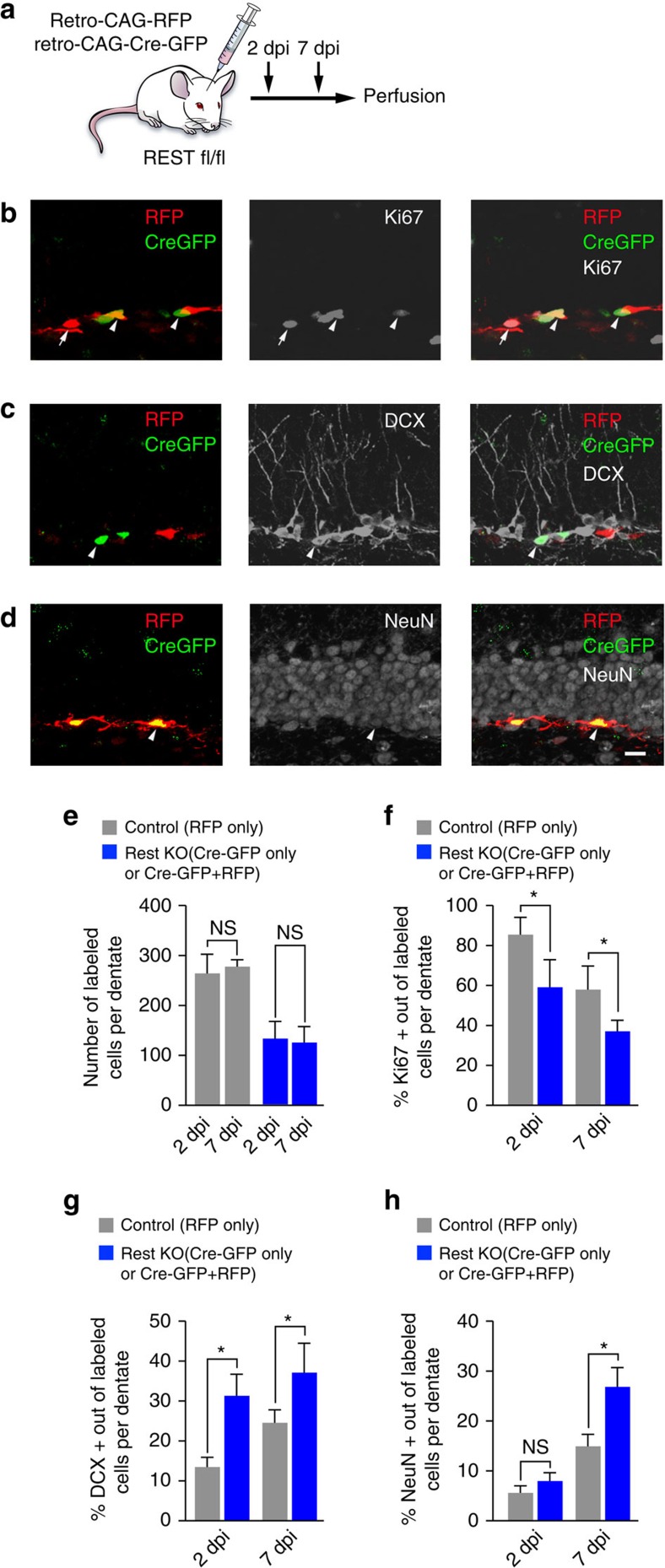
REST is required for maintenance of TAPs in adult hippocampus. (**a**) Retrovirus CAG-Cre-GFP and control CAG-RFP injections were performed in hippocampal dentate gyrus of 6–8 week-old REST flox/flox (fl/fl) mice in 1:2 ratio. (**b**) Confocal images showing RFP+Ki67+ (arrow) control TAP cell and RFP+GFP+Ki67+ (arrowhead) or GFP+Ki67+ (arrowhead) REST knockout TAP cells at 7 dpi in REST fl/fl. (**c**,**d**) Confocal images showing control RFP+ only and knockout GFP+ only or RFP+GFP+ cells with DCX+ immature neurons (arrowhead), (**c**) or NeuN+ mature neurons (arrowhead), (**d**) cells at 7 dpi in REST fl/fl. (**e**) Quantification of the total number of control RFP+ only and knockout GFP+ only or RFP+GFP+ cells per dentate gyrus at 2 dpi and 7 dpi. (**f**) The number of Ki67+ TAPs with control RFP+ only and knockout GFP+ only or RFP+GFP+ cells with at 2 dpi and 7 dpi. (**g**,**h**) The number of mCh+DCX+ immature neurons (**g**) and mCh+NeuN+ mature neurons (**h**) at 2 dpi and 7 dpi in control RFP+ only and knockout GFP+ only or RFP+GFP+ cells. For all quantifications, data are plotted as the mean±s.e.m. (*P≤0.05 and ns, not significant). For all quantifications, data are plotted as the mean±s.e.m. (*P≤0.05 and ns, not significant). Experiments were analysed for statistical significance using Kruskal–Wallis nonparametric test, which included Dunn's multiple comparison test and generated exact *P* values. Scale bar in **d**, 20 μm. At least 4–5 mice of REST fl/fl genotype 6–8 week old mice were used.

Since enhanced TAP proliferation due to REST deletion is likely to reflect precocious QNP activation, to address the cell-autonomous role of REST in TAPs, we delivered a retrovirus expressing CAG-Cre-GFP together with a retroviral CAG-RFP control to specifically infect proliferating cells in REST fl/fl mice (Fig. 2a). We found that the dentate gyrus of REST fl/fl mice injected with Cre-encoding retrovirus contained a decreased percentage of Ki67+GFP+ or Ki67+GFP+RFP+ REST KO TAPs compared with Ki67+RFP only control TAPs at both 2 and 7 dpi (Fig. 2b,f). This observation is consistent with the cell-autonomous requirement of REST to maintain TAP proliferation. Further examination of control and REST KO cells with neuronal differentiation markers revealed an increased percentage of DCX+GFP+ or DCX+GFP+RFP+ REST KO immature neurons at 2 dpi and 7 dpi and NeuN+GFP+ or NeuN+GFP+RFP+ REST KO mature neurons at 7 dpi (Fig. 2c,d,g,h). Taken together, these results suggest that REST has a cell-autonomous role to maintain quiescence in QNPs and proliferation in TAPs, which is essential to preserve the adult neural stem cell pool and prevent premature differentiation into immature and mature neurons.

### *In vitro* model of quiescent neural stem cells

As REST is a transcriptional repressor of a large battery of coding and non-coding genes required for neuronal function^29^,^30^,^31^,^32^, we hypothesized that REST maintains QNPs and TAPs by binding to its target genes. To identify REST target genes involved in quiescence maintenance and the transition to proliferation, we took advantage of cultured adult rat hippocampal neural stem cells (HCN cells) to obtain sufficient cell numbers for ChIP-seq and RNA-seq analysis. Consistent with previous studies, HCN cells were maintained in a highly proliferative state with FGF-2 treatment (TAP conditions) and could be induced to quiescence with addition of BMP4 (induced QNP conditions or iQNPs) (Supplementary Fig. 3a,b; refs 13, 15, 33, 34, 35, 36, 37, 38). Immunostaining for Ki67 and 5-bromo-2deoxyuridine (BrdU) incorporation revealed that the HCN cells in TAP conditions were highly proliferative compared with cells in iQNP conditions (Supplementary Fig. 3c,d,f,h). Furthermore, PI-flow cytometry revealed that significantly fewer cells were in G0-G1 phase in TAP conditions compared to cells in iQNP conditions, but more cells were in S-phase of cell cycle in TAP conditions compared to cells in iQNP conditions (Supplementary Fig. 3g), consistent with the immunostaining results.

As treatment with BMP alone can induce neuronal and glial differentiation^39^, we next examined whether the quiescence induced by BMP4, together with FGF-2 is reversible by removing BMP4-containing medium and returning HCN cells to FGF-2-containing medium (TAP') (Supplementary Fig. 3a,b). After 6 days in TAP' conditions, HCN cells resumed proliferation at a rate comparable to that of control TAPs, as measured by PI-flow cytometry and Ki67 and BrdU staining. We observed that cells which reverted back to TAP' conditions had decreased G0-G1 phase of cell cycle and increased S phase compared to cells in iQNP conditions (Supplementary Fig. 3g), as well as increased Ki67+ and BrdU+ cells, similar to HCN cells continuously grown in TAP conditions (Supplementary Fig. 3c,d,f,h). To further confirm whether HCN cells under TAP' conditions are reversible, we treated TAP' cells with lineage-specific differentiation media and compared them to TAP cells treated with the same conditions. We determined HCN cells in TAP' conditions retained their multi-lineage potential and stained for markers of neurons (Tuj1), astrocytes (GFAP) and oligodendrocytes (RIP1) (Supplementary Fig. 3e,i). These results suggest that BMP-mediated quiescence in HCN cells is a reversible state, consistent with previous studies^13^,^33^,^34^.

### REST is required to maintain iQNPs and TAPs *in vitro*

**Figure 3:**
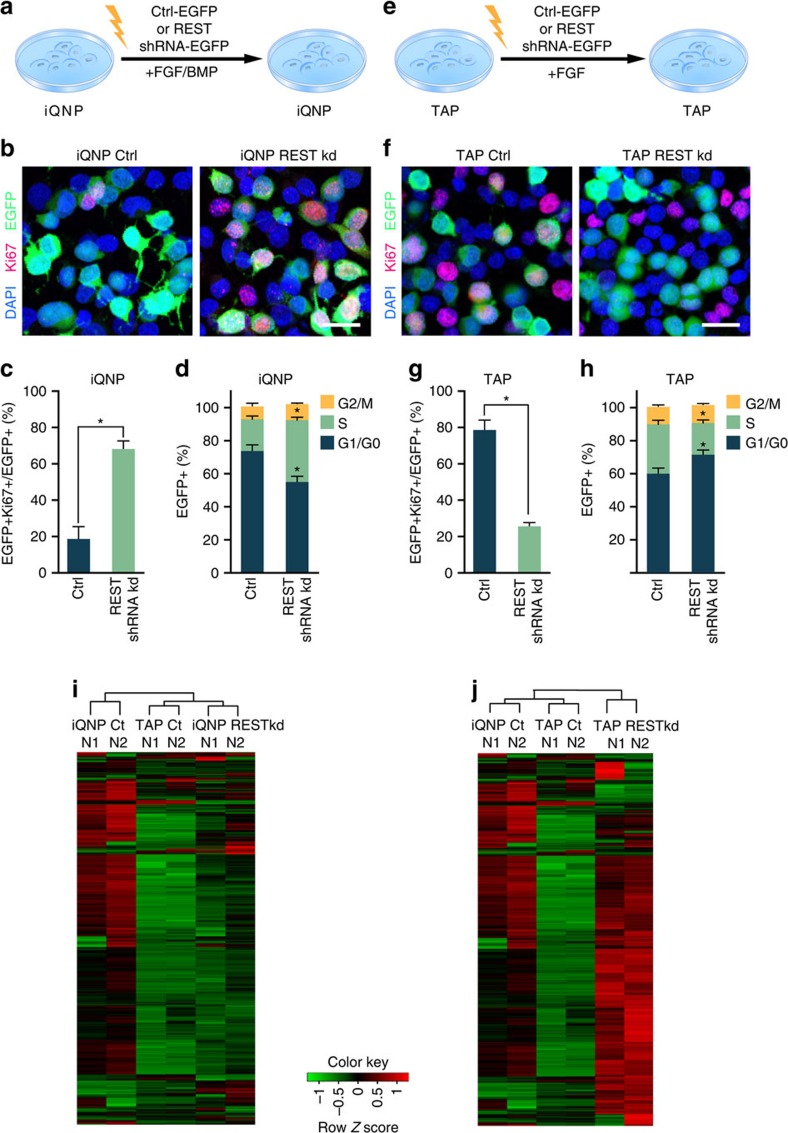
REST is required for maintenance of iQNP and TAPs *in vitro*. HCN cells in (**a**) iQNP conditions or (**e**) TAP conditions were electroporated with control EGFP or REST shRNA-EGFP vector. Immunofluorescent analysis of electroporated cells fixed at 2.5 days *in vitro* and stained with proliferation markers, (**b**) Ki67 (red) in iQNP conditions and (**f**) Ki67 (red) in TAP conditions. Quantification of electroporated (**c**) Ki67 in iQNP conditions and (**g**) Ki67 in TAP conditions as per cent of all electroporated cells (EGFP+). Cell cycle analysis by PI flow cytometry of electroporated HCN cells in (**d**) iQNP conditions and (**h**) TAP conditions. Heat map and hierarchial clustering of RNA-seq from electroporated control EGFP and REST shRNA-EGFP in iQNP (**i**) and TAP (**j**). For all quantifications, data are plotted as the mean±s.e.m. (*P≤0.05 and ns, not significant). Experiments were analysed for statistical significance using an unpaired, two-tailed Student's *t*-test. Scale bars in **b**,**f**, 20 μm. All experiments were performed at least three times independently. See also Supplementary Fig. 4.

To identify putative REST target genes in quiescence and proliferation, we first performed experiments to determine the requirement of REST in HCN cells *in vitro* (Fig. 3a,e). For HCN cells in iQNP conditions, we observed a marked increase in REST short hairpin RNA (shRNA)-EGFP electroporated cells that expressed Ki67 and incorporated BrdU relative to control EGFP electroporated cells in iQNP conditions (Fig. 3b,c; Supplementary Fig. 4b,c). These results were also supported with PI-flow cytometry cell cycle analysis that showed REST knockdown decreased the percentage of cells in G0-G1 phase and increased the percentage of cells in S-phase in iQNP conditions (Fig. 3d).

For HCN cells in TAP conditions, we found REST knockdown led to a decrease in cells that expressed Ki67 and incorporated BrdU relative to control electroporated cells (Fig. 3f,g; Supplementary Fig. 4e,f). Moreover, PI-flow cytometry cell cycle analysis showed REST knockdown increased the percentage of cells in G0-G1 and decreased percentage of cells in S-phase of cell cycle REST shRNA in TAP conditions (Fig. 3h). Altogether, these data suggest REST is required to maintain iQNPs and TAPs *in vitro*, similar to its role *in vivo*.

To provide additional evidence that REST is required to maintain both iQNPs and TAPs and to begin to identify REST target genes involved in this process, we performed RNA-seq analysis in HCN cells electroporated with a REST shRNA vector to knockdown REST compared with HCN cells electroporated with an empty shRNA vector (control) (Fig. 3i,j; Supplementary Data 1,2). We found gene expression in iQNPs after REST knockdown clustered more with gene expression in TAP conditions compared with iQNP conditions, consistent with REST being required to maintain quiescence (Fig. 3i). Interestingly, gene expression in TAPs after REST knockdown did not cluster with either gene expression in TAP conditions or iQNP conditions, suggesting that loss of REST in TAPs may be turning on a differentiation program (Fig. 3j). To examine this possibility, we stained HCN cells with the neuronal marker Tuj1+ and observed increased percentage of EGFP+Tuj1+ in cells electroporated with REST shRNA-EGFP compared with control electroporated cells, consistent with premature neuronal differentiation due to loss of REST in TAPs (Supplementary Fig. 4g,h). These results demonstrate REST is required to maintain both the quiescence and proliferation states to prevent premature differentiation into neurons, and consequently the identification of REST target genes in HCN cells is expected to define new REST targets important in regulating the adult neural stem cell pool.

### Identification of REST-binding sites in iQNPs and TAPs

**Figure 4:**
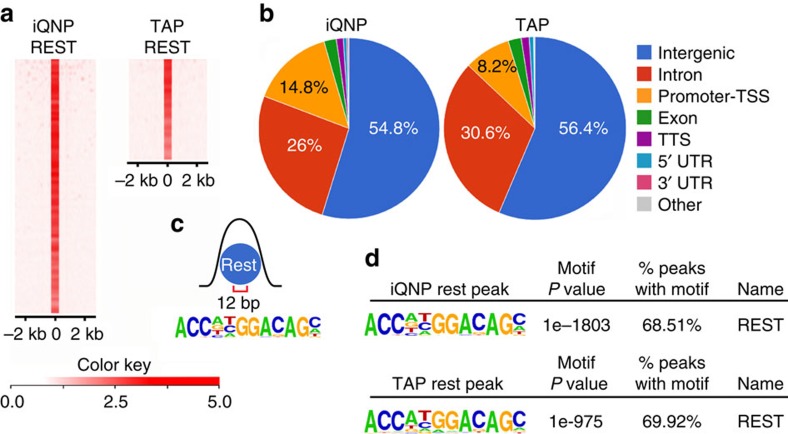
Identification of REST-binding sites of HCN cells in iQNP and TAP conditions. (**a**) Heat maps of REST ChIP-seq peaks in iQNP and TAP conditions. (**b**) Genomic distribution of REST binding categorized based on associated type of genomic region in iQNP and TAP conditions. (**c**) REST-binding motif. (**d**) *De novo* identified REST motif by HOMER in iQNP and TAP conditions. See also Supplementary Fig. 5.

To characterize the regions bound by REST in quiescent and proliferating HCN cells, we performed genome-wide ChIP-seq using multiple REST antibodies against the C-terminal domain of REST^40^,^41^,^42^. HOMER analysis identified 1,775 and 778 REST bound sites overlapping between multiple REST antibodies in iQNP and TAP conditions, respectively (Fig. 4a; Supplementary Fig. 5b,c; Supplementary Data 3,4).

To determine the genome-wide distribution of REST bound peaks in iQNP and TAP conditions, we used ChIPseek analysis^43^. The global binding pattern was similar in iQNP and TAP conditions, with more of the peaks mapping to intergenic regions compared with intron/promoter-transcription start site (TSS) +/−1 kb combined regions (Fig. 4b). Next, we searched for transcription factor motifs among REST bound sites that mapped within +/−10 kb of promoter-TSS^44^. HOMER *de novo* motif algorithm identified the known REST-binding motif also known as the repressor element 1 (RE1) site (MA0138.2 Jaspar), or its reverse complement, in 70% of sites in iQNP and TAP conditions (Fig. 4c,d; Supplementary Data 5,6). These results suggest that in both iQNP and TAP conditions, REST binds to its own consensus DNA-binding site.

### REST binds distinct target genes in iQNPs and TAPs

**Figure 5:**
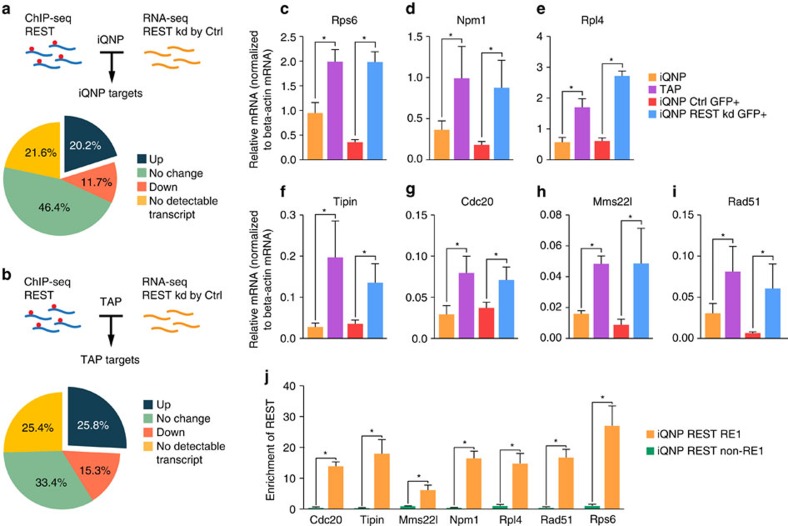
Integration of ChIP-seq and RNA-seq data sets to identify direct REST targets in iQNPs and TAPs. (**a**,**b**) Distribution of gene expression changes associated with REST-binding sites annotated to +/−10 kb of gene TSS and RNA-seq data in REST knockdown and control electroporation in iQNP (**a**) and TAP (**b**) conditions. (**c**–**i**) To validate RNA-seq data in iQNP, qPCR was performed on HCN cells in non-electroporated iQNP and TAP cells, and those electroporated with a control EGFP or REST shRNA-EGFP vector in iQNP. qPCR validation of iQNP REST candidate DNA replication genes (Cdc20, Tipin, Mms22l and Rad51) and ribosome biogenesis genes (Npm1, Rpl4 and Rps6). (**c**–**i**) To show REST-dependent gene expression of targets, qPCR was performed on HCN cells electroporated with a control EGFP or REST shRNA-EGFP vector in iQNP conditions for non-neuronal targets. (**j**) To validate ChIP-seq data in iQNP and TAP conditions ChIP-qPCR was performed. ChIP-qPCR validation of iQNP REST candidate DNA replication genes (Cdc20, Tipin, Mms22l and Rad51) and ribosome biogenesis genes (Npm1, Rpl4 and Rps6). For all quantifications, data are plotted as the mean±s.e.m. (*P≤0.05 and ns, not significant). Experiments were analysed for statistical significance using an unpaired, two-tailed Student's *t*-test. All experiments were performed at least three times independently. See also Supplementary Fig. 5; Supplementary Fig. 6; Supplementary Fig. 7.

To identify candidate direct targets of REST, we reasoned that because REST is a transcriptional repressor^25^,^40^, combining REST ChIP-seq analysis with RNA-seq of genes deregulated by REST knockdown will identify direct targets that are bound and repressed by REST. We, therefore, superimposed the RNA-seq data set of REST knockdown and control vector transduced iQNPs and TAPs (Fig. 3i,j) with iQNP ChIP-seq and TAP ChIP-seq (Fig. 4a,b), respectively, to identify REST-dependent direct targets in iQNP and TAP conditions (Fig. 5a,b). Among the candidate REST targets in iQNP conditions, 20.2% of genes were upregulated in REST knockdown iQNPs compared with control electroporation (iQNP targets) (Fig. 5a). Conversely, among the candidate REST targets in TAP conditions, 25.8% of genes were upregulated in REST knockdown in TAPs compared with control electroporation (TAP targets) (Fig. 5b). Comparison of iQNP targets with TAP targets revealed a set of 79 unique iQNP targets, 19 unique TAP targets and 44 REST targets common in both iQNPs and TAPs (Supplementary Table 1; Supplementary Data 7–9).

Next, to determine the mechanism by which REST regulates distinct iQNP and TAP targets, we hypothesized that REST binds unique and common iQNP and TAP targets based on: (1) differential REST binding, (2) REST motif variations and/or (3) enrichment of other transcription factor motifs. To examine differential REST binding at unique and common iQNP and TAP targets, we performed ngs.plot analysis^45^. As expected, there was similar enrichment of REST binding at common targets in iQNP and TAP conditions (Supplementary Fig. 6a). For unique iQNP targets, there was significantly more enrichment of REST binding in iQNP relative to TAP conditions (Supplementary Fig. 6b). Surprisingly, REST binding on unique TAP targets was comparable in both iQNP and TAP conditions (Supplementary Fig. 6c). To examine the possibility that variation in the REST motif or presence of other motifs contribute to REST binding of unique and common iQNP and TAP targets, we performed HOMER *de novo* motif analysis. REST motif emerged as the prominent motif in 55.3% of unique iQNP targets, 68.9% of unique TAP targets and 62.2% of common targets (Supplementary Fig. 6d–f). The canonical REST-binding motif was found in 55.32% of unique iQNP targets, but was slightly different (designated as REST*) in unique TAP and common iQNP and TAP targets (Fig. 4d; Supplementary Fig. 6d–f). Moreover, we observed the presence of other motifs in unique iQNP and TAP targets; for example, the GFX and MAF-A motifs in unique iQNP targets and the MAF-A, DUX, GLI3 and MECOM motifs in unique TAP targets (Supplementary Fig. 6e,f). Altogether, these results suggest that enrichment of REST, a variable REST/RE1 motif, and the combination of other transcription factor motifs may be involved in the regulation of REST-dependent target genes relevant to the quiescent and proliferative states.

To survey the biological significance of these direct REST targets in iQNPs and TAPs, we used Protein ANalysis THrough Evolutionary Relationships (PANTHER)^46^. As expected, genes related to neuronal function such as ‘cation transport/ion transport', ‘synaptic transmission' and ‘nervous system development' appeared as significant GOs in iQNPs and TAPs regardless of whether the genes were unique or common targets, consistent with the known role of REST as a neuronal gene repressor (Supplementary Table 1, Supplementary Data 10–12). Interestingly, we identified unique iQNP targets related to ‘ribosome biogenesis', ‘translation', and ‘cell cycle', suggesting that these gene networks may have a role in controlling the quiescent state.

### REST regulates ribosome biogenesis and cell cycle genes

As little is known about the role of REST in ribosome biogenesis and cell cycle progression, we selected seven representative unique iQNP targets from ‘ribosome biogenesis' and ‘cell cycle' GOs for further validation and functional studies (Supplementary Table 1; Supplementary Fig. 7a–g). Specifically, we focused on nucleophosmin 1 (Npm1), ribosomal protein S6 (Rps6), ribosomal protein L4 (Rpl4), cell division cycle 20 (Cdc20), methyl methanesulfonate-sensitivity protein 22-like (Mms22l), rad51 recombinase (Rad51) and timeless interacting protein (Tipin).

First, to validate our RNA-seq data, we performed quantitative PCR (qPCR) in iQNPs with and without REST knockdown (Fig. 5c–i). We observed the mRNA expression of all 7iQNP targets were derepressed after REST knockdown in iQNPs, consistent with REST-dependent repression of these genes (Fig. 5c–i). We also performed qPCR in iQNPs and TAPs and found all seven iQNP targets were downregulated in iQNPs compared with TAPs, suggesting these genes played a role in the transition between iQNPs and TAPs (Fig. 5c–i). Next, to validate our ChIP-seq data, we confirmed REST binding with ChIP-qPCR of all seven iQNP targets and depletion of binding of select targets in REST knockdown samples compared with controls (Fig. 5j, Supplementary Fig. 5d,e). Altogether these results suggest that REST-dependent repression of ribosome biogenesis and cell cycle genes may be an important part of the regulatory network to maintain iQNPs and TAPs.

**Figure 6:**
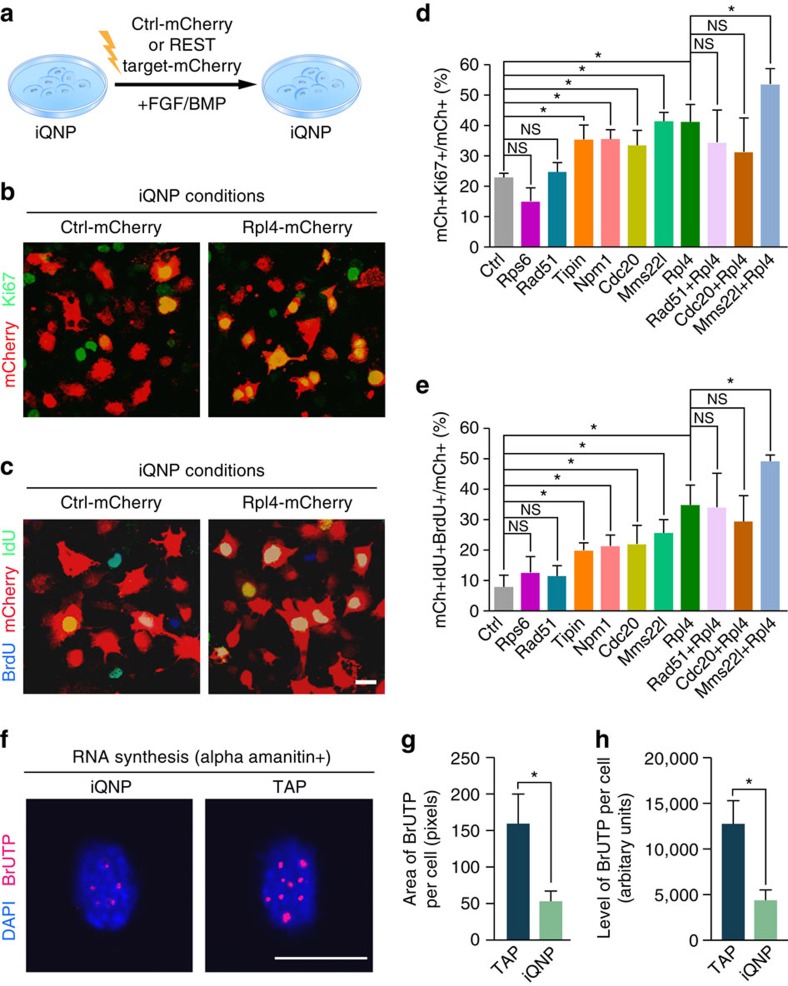
Role of REST target ribosome biogenesis and cell cycle genes in iQNP conditions. HCN cells in (**a**) iQNP conditions were electroporated with control lenti-hGFAP-IRES-mCh or lenti-hGFAP-cDNA-IRES-mCh overexpression vector. (**b**,**c**) Immunofluorescent analysis of electroporated cells fixed at 3 days *in vitro* and stained with proliferation markers, Ki67 (green) (**b**) or pulsed first with BrdU (blue) at 2 days followed by IdU (green) (**c**) pulse at 3 days and fixed at 3 days in iQNP conditions. (**d**,**e**) Quantification of electroporated Ki67 (**d**) or BrdU+IdU+ (**e**) in iQNP conditions as percent of mCh+ cells. (**f**–**h**) Immunofluorescent analysis BrUTP+(red) DAPI+(blue) (**f**) and quantification of BrUTP labelling area (**g**) and intensity (**h**) in iQNP and TAP conditions. For all quantifications, data are plotted as the mean±s.e.m. (*P≤0.05 and ns, not significant). Experiments were analysed for statistical significance using an unpaired, two-tailed Student's *t*-test. Scale bar in **c**,**f**, 20 μm. All experiments were performed at least three times independently. See also Supplementary Fig. 8.

Since these iQNP REST targets were downregulated in iQNPs compared with TAPs, we overexpressed these genes in iQNP conditions to evaluate their functional roles (Fig. 6a). To overexpress select REST quiescence targets in HCN cells in iQNP conditions, we cloned complementary DNAs (cDNAs) encoding each gene into a lentiviral expression vector driven by a hGFAP promoter and containing an IRES-mCherry fluorescent reporter. We confirmed overexpression of select ribosome biogenesis and cell cycle genes (by ∼3 to 57 fold) in electroporated HCN cells compared with cells electroporated with a control lenti-hGFAP-IRES-mCh vector (Supplementary Fig. 8a). Interestingly, we observed overexpression of Cdc20, Tipin, Mms22l, Npm1 or Rpl4, but not Rad51 or Rps6, increased proliferation of HCN cells in iQNP conditions, as indicated by an increased colocalization of mCh+ cells with Ki67 expression (Fig. 6b,d).

To further evaluate the effect of REST quiescence target genes in cell cycle progression, we performed pulse-chase-pulse label with BrdU and iododeoxyuridine (IdU) to detect successive progression through S phase. Similar to higher Ki67 expression, we found overexpression of REST quiescence targets led to increased percentage of IdU+BrdU+ electroporated cells compared with control electroporated cells (Fig. 6c,e), suggesting that cells exited quiescence and progressed through multiple cell cycles. Interestingly, combining ribosome biogenesis and cell cycle genes, Rpl4 and Mms22l, respectively, but not Rad51 and Rpl4 or Cdc20 and Rpl4 showed an additive effect in activation of iQNPs (Fig. 6b–e). These results suggest REST has a role in maintaining quiescence *in vitro*, by repressing ribosome biogenesis and cell cycle genes. Moreover, these results also indicate the cooperation between ribosome biogenesis and cell cycle genes in regulation of quiescence.

The previous finding that overexpression of Npm1 and Rpl4 promotes HCN cells to exit quiescence suggests that ribosome biogenesis may contribute to the reduction of protein synthesis, consistent with emerging work that regulation of ribosome biogenesis and protein synthesis is critical for stem cell homeostasis^47^. Since ribosomes consist of ribosome proteins and ribosomal RNA (rRNA), two integral components of ribosome biogenesis, we next evaluated rRNA synthesis in iQNPs compared with TAPs. We performed the bromouridine-triphosphate (BrUTP) pulse-labelling assay in the presence of α-amanitin, an inhibitor of RNA polymerases II and III, to compare RNA polymerase I mediated rRNA synthesis between HCN cells in iQNP and TAP conditions. Interestingly, we found a significant decrease in BrUTP incorporation—both in the area occupied by BrUTP puncta per cell and the intensity level per cell—in iQNPs relative to TAPs (Fig. 6f–h; Supplementary Fig. 8b–e). This suggests that a reduction in rRNA transcription contributes to lower overall ribosome biogenesis in iQNPs compared with TAPs. Altogether these results suggest that REST-dependent repression of ribosome biogenesis genes such as Npm1 or Rpl4 may be an important part of the gene expression program to maintain neural stem cell quiescence.

### Role of REST quiescence targets *in vivo*

**Figure 7:**
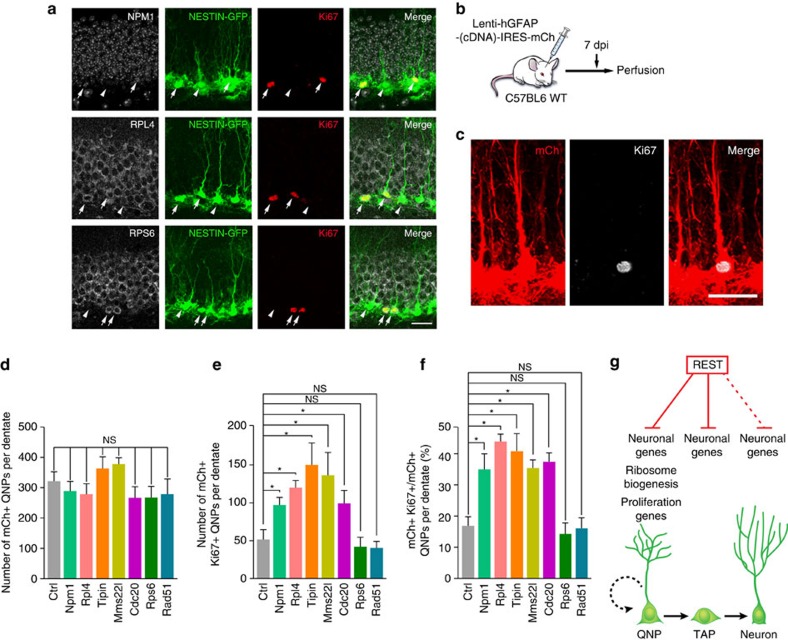
REST target ribosome biogenesis and cell cycle genes promote activation of QNPs in adult hippocampus. (**a**) Confocal images of adult Nestin-GFP+ hippocampus mouse sections. Arrow indicates a Nestin-GFP with or without radial process (green), Ki67+ (red), and Npm1+ or Rpl4+ or Rps6+ (grey) triple-labelled activated QNP and TAP cell, respectively. Arrowhead indicates a Nestin-GFP+ with radial process (green), Ki67- (red) QNP cell where expression of Npm1- or Rpl4- or Rps6- (grey) was barely detectable. One representative image from three independent 6–8 week-old Nestin-GFP transgenic mice are shown. (**b**) Lentivirus hGFAP-(cDNA)-IRES-mCh injections were performed in hippocampal dentate gyrus of 6–8 week old WT C57BL6 mice. (**c**) Confocal images showing mCh+Ki67+ activated QNP and mCh+Ki67- QNP at 7 dpi in WT C57BL6 mice. (**d**–**f**) Quantification of the total number of mCh+ QNP cells per dentate gyrus (**d**) and the number and percentage of mCh+Ki67+ activated QNP cells at 7 dpi (**e**,**f**). (**g**) Model of REST regulation of gene networks in QNPs, TAPs and neurons. For all quantifications, data are plotted as the mean±s.e.m. (*P≤0.05 and ns, not significant). For all quantifications, data are plotted as the mean±s.e.m. (*P≤0.05 and ns, not significant). Experiments were analysed for statistical significance using Kruskal–Wallis nonparametric test, which included Dunn's multiple comparison test and generated exact *P* values. Scale bar in **a** and **c**, 20 μm.

We identified REST target genes in HCN cells, which is a starting point towards elucidating the *in vivo* function of REST target genes. To begin to examine the *in vivo* role of REST target genes, we analysed the expression pattern of REST target ribosome biogenesis genes in QNPs and TAPs using Nestin-GFP transgenic mice. We found that Npm1, Rpl4 and Rps6 are expressed in activated QNPs (radial morphology Nestin-GFP+Ki67+) and TAPs (non-radial morphology Nestin-GFP+Ki67+), but to a lesser extent in QNPs (radial morphology Nestin-GFP+Ki67−) (Fig. 7a). This is consistent with their role in activating iQNPs *in vitro*. Next, to test the function of REST target genes *in vivo*, we injected C57Bl6 WT mice with hGFAP-cDNA-IRES-mCh lentiviruses overexpressing individual REST target genes (Fig. 7b). At 7 dpi, we confirmed the total number of mCh+ QNPs per dentate were unchanged in injected mice (Fig. 7d), consistent with effective virus targeting. We found that WT mice injected with hGFAP-cDNA-IRES-mCh lentivirus showed an increased number and percentage of mCh+Ki67+ activated QNPs cells relative to WT mice injected with empty vector mCh+ lentivirus at 7 dpi for, Rpl4, Tipin, Mms22l and Cdc20, but not for Rps6 and Rad51 (Fig. 7c–f), similar to the effect of these genes *in vitro.* Altogether these results demonstrate that REST controls the transition of QNPs to activated QNPs and TAPs *in vivo* by repressing select ribosome biogenesis and cell cycle genes.

## Discussion

Despite the tremendous interest and therapeutic potential of newborn neurons, we still have incomplete understanding of the mechanisms that control activation of neural stem cell quiescence and transition to proliferative stages. In this study, we provide several lines of evidence suggesting that REST is required in both quiescent and proliferating neural progenitors to bind and regulate distinct gene programs. We also show that genome-wide transcriptome analysis in adult hippocampal neural stem cells can reveal important insight into the biology of cellular quiescence.

In previous work, we conditionally deleted REST from QNPs and TAPs in adult hippocampus and saw an initial increase in activated QNPs and newborn neuron differentiation followed by an eventual decrease in activated QNPs and newborn neurons^25^. Despite these early findings suggesting REST may have a central role in maintaining the adult neural stem cell pool, the original Nestin-CreER^T2^ transgene used to conditionally delete REST was expressed in both QNPs and TAPs and not specific to QNPs. Now, using a cell-specific viral approach to precisely delete REST in adult hippocampal QNPs, we show deletion of REST in QNPs results in the loss of quiescence and rapid transition to proliferation, consistent with REST playing a cell-autonomous role to preserve the quiescent state and prevent precocious activation *in vivo*. We also show REST is required cell-autonomously in TAPs to maintain proliferation and prevent premature differentiation into immature and mature neurons. Consistent with the role of REST to maintain both QNPs and TAPs, REST knockdown *in vitro* also leads to loss of quiescence, decreased TAP proliferation and enhanced neuronal differentiation. Taken together, our findings suggest REST has a context-dependent role to maintain quiescence in QNPs and proliferation in TAPs, ultimately to prevent premature differentiation into adult-born neurons. Next, we propose hypotheses to explain the mechanism by which REST controls quiescence and transition to proliferative stages.

ChIP-seq and RNA-seq analysis revealed the majority of REST targets in quiescence and proliferative stages were neuronal genes (for example, cation transport/ion transport, synaptic transmission, nervous system development), consistent with the important role of REST as a repressor of neuron-specific genes^40^,^48^,^49^. It is possible that one of the major roles of REST in iQNP and TAP conditions is to repress the neuronal lineage program essential for the maintenance of the undifferentiated state. Supporting this hypothesis, we observed knockdown of REST in HCN cells was sufficient to upregulate Tuj1+ neurons. It will be interesting to determine if overexpression of distinct REST neuronal target genes in iQNPs or TAPs can directly promote neuronal differentiation in future work. Recent work also points to the role of the proneural transcription factor Ascl1 to regulate the proliferation of QNPs^19^,^50^. Interestingly, a paper suggests REST may directly bind and control the expression of Ascl1^42^. Although we did not observe direct binding of REST within +/−10 kb of the Ascl1 genomic locus in HCN cells in iQNP conditions, REST may cooperate with additional factors to regulate the quiescent state. Consistent with this idea, we identified the GFX transcription factor motif that overlapped with RE1 sites in a subset of iQNP targets.

Although we do not yet understand the functional significance of differential neuronal gene expression in iQNP and TAP conditions or whether REST repression of distinct subsets of neuronal genes is strictly required for the transition between QNPs to TAPs, we speculate that REST recruitment of co-repressors such as Sin3A or CoREST^51^ and/or polycomb repressor complexes ½ (ref. 52) may establish specific epigenetic marks, such as histone methylation H3K4Me3 and H3K27Me3 to control differential neuronal gene expression in QNPs and TAPs. Future work to explore the presence of epigenetic histone modifications at REST target neuronal genes may reveal additional insight regarding the molecular nature by which REST controls gene expression in adult hippocampal neural stem cells.

While the majority of REST target genes in iQNP and TAP conditions were neuronal genes, strikingly, REST targets unique to quiescence belonged to ribosome biogenesis and cell cycle. Since proliferative and metabolic silencing are hallmarks of quiescence^53^ and emerging work suggests the importance of regulating protein synthesis and metabolic state in stem cells^54^, it is exciting to speculate whether REST can regulate both the proliferative and metabolic state in QNPs and entry into cell cycle, cell growth and protein synthesis through binding specific gene targets. Based on the finding that Npm1, a known regulator of processing pre-rRNA to mature rRNA which can be assembled with ribosomal proteins to form the ribosome for protein synthesis^55^, was bound and repressed by REST in iQNP conditions, and rRNA synthesis was decreased in iQNP conditions as indicated by the BrUTP assay, a possible mechanism by which REST regulates rRNA processing in iQNPs is through regulating Npm1 levels. Furthermore, REST also represses Rpl4, a ribosome subunit protein highly expressed during neurogenesis^56^. This suggests that REST may repress ribosome biogenesis and thereby protein synthesis by repressing the expression of Rpl4 which is needed to make the ribosome. Consistent with the important role of ribosome biogenesis genes in regulating quiescence, overexpression of Npm1 or Rpl4 was sufficient to induce proliferation of QNPs. Moreover, overexpression of cell cycle genes Mms22l, Tipin or Cdc20 also induced proliferation of QNPs, suggesting cooperation between multiple REST target genes. We present the following model (Fig. 7g) to illustrate how REST may control quiescence and the transition to proliferation and differentiation through coordinated regulation of neuronal, ribosome biogenesis and proliferation genes.

Our work identifies distinct REST target genes in QNPs and TAPs. However, the mechanism underlying how REST differentially regulates target genes remains to be investigated. As REST mRNA and protein levels are not different between iQNP and TAPs (data not shown), we still lack an explanation regarding the differential binding of REST in unique iQNP targets (Supplementary Fig. 6b). One possibility is the REST peaks on unique iQNP targets have the canonical REST-binding motif compared with the slightly altered REST*-binding motifs on unique TAP and common iQNP and TAP targets, which could potentially mediate differential REST binding on unique iQNP targets (Supplementary Fig. 6a–c). For unique TAP targets with equal binding of REST in both iQNPs and TAPs, we still do not fully understand how these genes are only derepressed in TAP conditions. One possibility is the presence of other transcription factor motifs, such DUX, Gli3 and MECOM, which might recruit activators in TAP but not in iQNP conditions. Future studies are needed to address whether REST interacts with other transcription factors to control distinct target gene expression and whether REST-binding partners have crucial roles in QNPs and TAPs. Taken together, our data highlights the context-dependent regulation of REST in adult neural stem cells, from quiescence to proliferation, before differentiating into mature neurons.

## Methods

### Animals and stereotaxis of brain

Animals were treated humanely and protocols were approved by the Institutional Animal Care and Use Committee of University of Texas Southwestern Medical Center at Dallas, USA. For *in vivo* expression analyses of REST, Nestin-GFP transgenic and WT (C57BL6) mice were used with the following combination of cell type specific markers and assessment of cell type morphology. We confirmed by western blot analysis that our ‘homemade' antibody (REST14) used in immunostaining detects full-length REST (Supplementary Fig. 1g). Since GFAP labels both QNPs and astrocytes, we also used cell morphology to identify GFAP+ QNPs with a radial process. GFAP+Sox2+ and GFAP+Ki67+ cells with radial process were quantified as QNPs and activated QNPs, respectively. To distinguish TAPs from activated QNPs, we assessed the morphology of Nestin-GFP+ cells in transgenic reporter mice^57^ and expression of Ki67. Nestin-GFP+Ki67+ with round soma and no radial process were quantified as TAPs. Nestin-GFP-Ki67+ cells, DCX+ cells and NeuN+ cells were quantified as neuroblasts, immature neurons and mature neurons, respectively. For *in vivo* REST deletion, lentivirus hGFAP-Cre-p2A-mCh was injected into the dentate gyrus (coordinates from bregma AP −2.0, ML+−1.5, DV-2.5) by stereotaxis in REST fl/fl and WT (REST+/+) mice. One or both hemispheres of the brains, from female and male, 6–8 week mice were used for all experiments. REST fl/fl or REST wild type (WT) littermate control mice were injected with the same titer of hGFAP-Cre-p2A-mCh lentivirus into the right and left dentate gyrus (Fig. 1a). At 2 days post infection (dpi) in control mice, we performed immunohistochemical staining to confirm the specificity of mCh expression upon infection of hGFAP-Cre-p2A-mCh lentivirus. All cells in the subgranular zone expressing mCh from viral transfection, GFAP alone or with Sox2 and with QNP cell morphology (radial process) were quantified as QNP cells. Activated QNP (aQNP) cells were the subset of these cells that exhibited radial morphology and co-labelled with Ki67. TAPs (non-radial, round soma) in subgranular zone generated from asymmetric division of aQNPs cells were quantified as mCh+, GFAP- and Ki67+ (Fig. 1a–g). Only TAPs found directly adjacent to an aQNP were counted (Fig. 1a–g). mCh+ DCX+ cells and mCh+ NeuN+ cells were quantified as immature neurons, and mature neurons, respectively (Fig. 1h–l). As expected, triple labelling of GFAP, Sox2 and mCh, and morphological assessment of mCh+ cells revealed that the majority of the mCh expressing cells were GFAP+Sox2+ QNPs (54.6%) or mature astrocytes (25.4%), while only a small number of mCh expressing cells were GFAP-Sox2+ TAPs (<5%) and none were DCX+NeuN+ neurons (Supplementary Fig. 2a,c,d,e,f). Moreover, we confirmed that some of the mCh+ cells were GFAP+Ki67+ activated QNPs (15.8%) (Supplementary Fig. 2b,d,e,f). To determine the efficiency of REST deletion, we performed fluorescence-activated cell sorting (FACS) of mCh+ and mCh-negative cells from REST fl/fl mice infected with hGFAP-Cre-p2A-mCh lentivirus at 2 dpi. First, we confirmed that the total number of mCh+ QNP cells were comparable in the dentate gyrus of injected REST fl/fl or WT control mice, consistent with an equal volume of the same virus being injected in the same site in both groups of mice (Fig. 1c). We then counted the number of mCh+Ki67+ QNP cells in REST fl/fl and WT mice.

For *in vivo* REST deletion in TAPs only, retroviruses control CAG-RFP and CAG-Cre-GFP were injected in 2:1 ratio into the dentate gyrus (coordinates from bregma AP −2.0, ML+−1.5, DV-2.5) by stereotaxis in REST fl/fl mice. One or both hemispheres of the brains, from female and male, 6–8 week mice were used for all experiments. For these experiments, REST fl/fl mice were injected with a 1:2 mixture of CAG-Cre-GFP and CAG-RFP to infect and obtain control (RFP only) and REST knockout (KO) cells (GFP only or GFP+RFP+) (Fig. 2a). First, we confirmed that the total number of control and REST KO cells, respectively, were comparable in the dentate gyrus of injected REST fl/fl at 2 dpi and 7 dpi, consistent with equivalent virus titer injected in all mice (Fig. 2e). Next, we performed immunohistochemical for Ki67 to determine the proliferation state of RFP+ control and GFP+ or GFP+RFP+ REST KO cells (Fig. 2b). All cells in the subgranular zone expressing RFP+ only (control TAPs) and RFP+GFP+ or GFP+ only (knockout TAPs) were labelled and quantified using proliferation marker Ki67 or immature neuron marker DCX or mature neuron market NeuN at 2 dpi and 7 dpi.

For *in vivo* gain of function experiment or candidate gene overexpression (Npm1, Rpl4, Tipin, Mms22l, Cdc20, Rps6 and Rad51) cDNA from Dharmacon plasmids (more information available on request) were cloned into lentivirus hGFAP-cDNA-IRES-mCh, packaged into lentivirus and were injected into the dentate gyrus (coordinates from bregma AP −2.0, ML+−1.5, DV-2.5) by stereotaxis in WT C57BL6 (ENVIGO) mice. Empty lentivirus, hGFAP-IRES-mCh was used as control. One or both hemispheres of the brains, from female and male, 6–8 week mice were used for all experiments. All cells in the subgranular zone expressing mCh from viral transfection with QNP cell morphology (radial process) were quantified as QNP cells. Activated QNP (aQNP) cells were the subset of these cells that exhibited radial morphology and co-labelled with Ki67. Only QNPs and aQNPs were counted (Fig. 7b–f).

### Immunohistochemistry of brain tissue

Immunohistochemistry was performed as previously described^25^. Briefly, animals were anaesthetized and transcardially perfused with 4% paraformaldehyde (PFA). Brains were dissected, post-fixed in 4% PFA overnight and both brain hemispheres were sectioned 30 μm thick on a freezing microtome. Free-floating or mounted sections were blocked with 3% normal donkey serum and 0.3% Triton-X. Sections were then incubated in primary followed by secondary antibodies diluted in blocking solution, 48 h at 4 °C and 2 h at room temperature (RT), respectively. In-between antibody incubation, sections were washed with Tris-buffered saline. Samples were imaged with a confocal laser-scanning microscope LSM 700 module from Zeiss and AIR from Nikon equipped with 4 laser lines (405, 488, 561 and 633nm) under 10 × , 20 × and 40 × objective lenses. Serial Z-stack images were obtained and collapsed to obtain a maximum intensity projection of lines and/or orthogonal crosshairs were displayed to indicate co-localization. A detailed list of antibodies is available in Supplementary Table 2.

### REST antibodies and western blotting

We generated two ‘homemade' REST antibodies (called REST14 and REST17) and used a commercial REST Ab (Millipore 17-641) for various experiments. The rabbit anti-mouse REST antibodies were raised against amino acids (N-terminal to C-terminal convention) 637 to 867 (REST14) and 867 to 1,097 (REST17) of REST (within REST exon 5) after expression in bacteria followed by glutathione-agarose affinity purification. Purified peptide was used for immunization of rabbits and collection of antiserum (Cocalico Biologicals, Inc). For *in vivo* expression analysis of REST in Supplementary Fig. 1, we used the REST14 Ab. Specificity of this antibody was confirmed by Western blotting (1:500) of extracts from REST fl/fl neurospheres after infection by Adenovirus-CAG-Cre-GFP (KO) or Adenovirus-CAG-GFP (WT). Standard western blot protocol was used. Briefly, cells were lysed in cell lysis buffer (50 mM Tris·HCl, pH 7.4, 150 mM NaCl, 1 mM EDTA and 1% Triton X-100) supplemented with a mixture of protease (Roche) and phosphatase inhibitors (Sigma). After protein quantification by the bicinchoninic acid colorimetric assay system (Thermo Scientific), protein was denatured by boiling in 2 × SDS loading buffer at 95 °C for 5 min. Around 40 ug of protein was loaded per well onto 7% SDS PAGE gels for western blotting. For loading control, we used mouse–anti-CREB (1:1,000). After electrophoresis in the 7% SDS PAGE gels, the proteins were transferred to adsorbent polyvinylidene difluoride membrane, blocked in 5% milk and incubated with primary antibody solution in blocking solution overnight with shaking at 4 °C. After washing with buffer, the membranes were incubated with horseradish peroxidase-conjugated (Cell Signaling) secondary antibodies. Immunoblots were developed with an ECL-plus kit (GE Healthcare).

### FACS and genomic PCR

To determine the recombination efficiency of lentivirus hGFAP-Cre-p2A-mCh at the REST fl/fl locus, we performed genomic PCR on FACS of mCh+ and mCh- cells in REST fl/fl mice. Genomic DNA amplification using primers (REST primer A 5′-gagccgtttcctgtgatggcattc-3′ and REST primer B 5′-ccagggttcagttctctacacccac-3′) flanking the floxed region of exon 4 from mCh+ cells revealed a 1.2 kb product, consistent with efficient Cre-mediated recombination compared with a 2.8 kB product from mCh-negative cells (Supplementary Fig. 2g). To determine specificity of REST antibodies used in ChIP-seq and ChIP-qPCR, HCN cells in iQNP condition electroporated with REST knockdown shRNA and control plasmid (details below) were FACS sorted and probed with the REST homemade antibodies and REST commercial antibodies by western blot as described above.

### *In vitro* quiescence model and electroporation

The adult hippocampal neural stem cell line (HCN cells) used was isolated and cloned from Fisher 344 rats and characterized in previous studies^13^,^35^,^36^. Briefly, HCN cells were cultured in DMEM:F12 supplemented with N2, glutamate and PSF in the presence of FGF2 (20 ng ml^−1^) (proliferation conditions) or FGF2 (20 ng ml^−1^) and BMP4 (50 ng ml^−1^) (quiescence conditions). For differentiation conditions, we treated HCN cells with retinoic acid (1 μM) and forskolin (5 μM) for 4 days (neuronal differentiation), leukemia inhibitory factor and BMP4 (50 ng ml^−1^ each) for 6 days (astrocyte differentiation) or insulin growth factor 1 (500 ng ml^−1^) (oligodendrocyte differentiation) for 4 days on poly-ornithine and laminin coated tissue culture plates.

Knockdown of REST in quiescent HCN cells and proliferating HCN cells was achieved by introducing shRNA plasmid cloned in a lentivirus pllu2g vector: 5′-tgtgtaacctgcagtaccatttcaagagaatggtactgcaggttacactttttt-3′ by electroporation. Electroporation was performed with an Amaxa electroporator at a ratio of 5 μg DNA per five million HCN cells. Downstream analyses were done 48 h after electroporation. For assessment of REST knockdown efficiency, REST mRNA level was measured with qPCR (qPCR method described below under RNA-isolation and RNA-seq/qPCR) in FACS sorted EGFP+ REST shRNA-EGFP electroporated cells and compared with control (pllu2g) FACS sorted EGFP+ electroporated cells in iQNP and TAP conditions. We used a shRNA (shRNA-EGFP) construct to knockdown REST in HCN cells by electroporation (Fig. 3a,e). We were able to induce a reduction of REST mRNA levels by 85% compared with control EGFP vector (Supplementary Fig. 4a,d).

Overexpression of REST non-neuronal targets in quiescent HCN cells was achieved by introducing cDNA from Dharmacon plasmids (more information available on request) and cloning them into a lentivirus hGFAP-IRES-mCh vector by electroporation. Electroporation was performed with an Amaxa electroporator at a ratio of 5 μg DNA per five million HCN cells (for example, 2.5 μg of gene and 2.5 μg control; 2.5 μg of gene 1 and 2.5 μg of gene 2; 2.5 μg of control 1 and 2.5 μg of control 2). Downstream analyses were done 72 h after electroporation. For assessment of overexpression efficiency, mRNA levels were measured with qPCR (qPCR method described below under RNA-isolation and RNA-seq/qPCR) in FACS sorted lenti hGFAP-cDNA-IRES-mCh+ electroporated cells and compared with control (lenti hGFAP-IRES-mCh) FACS sorted mCh+ electroporated cells in iQNP conditions.

### Flow cytometry

To determine cell cycle profile by propidium iodide (PI)-flow cytometry, HCN cells were extracted by Trypsin-EDTA (Sigma T3924), fixed in 2% PFA for 10 min at RT for GFP retention or directly permeabilized in 70% ethanol at 4 °C overnight. Cells were washed in phosphate buffered saline and stained with PI (Sigma P4864) in the presence of RNaseA (Invitrogen 12091) and their DNA content was measured on a FACScalibur flow cytometer (BD Bioscience) with 10,000 events per determination. Cell cycle profile was generated using Flowjo software (Tree Star Inc).

### Immunocytochemistry

Immunocytochemistry was performed on adherent HCN cells that were washed with phosphate buffered saline and fixed in 4% PFA for 20 min at RT. Cells were blocked and permeabilized (0.3% Triton X-100 and 3% Normal Donkey Serum in Tris-buffered saline) and stained with proliferation and differentiation markers. For BrdU studies, HCN cells were also incubated with BrdU (10 μM) for 1 h before fixing in 4% PFA and treated for 30 min in 2 N HCl at 37 °C for 30 min and neutralized with 0.1 M Borate for 10 min at RT before blocking. For BrdU and IdU, pulse chase pulse experiment, cells were incubated with BrdU (10 uM) for 1 h, replaced with media, and after 24 h cells were pulsed with IdU (10 uM). Then cells were fixed with 4% PFA and treated for 30 min in 2 N HCl at 37 °C for 30 min and neutralized with 0.1 M Borate for 10 min at RT before blocking. Cells were stained according to previous methods^25^. Stained cells were visualized using a Nikon TE2000-U inverted microscope and counted using CellProfiler software. A detailed list of antibodies is available in Supplementary Table1.

### ChIP-seq/ChIP-qPCR

HCN cells were crosslinked for 10 min in formaldehyde and crosslinking was stopped with 0.125 M glycine. Remaining steps of sonication and ChIP of sonicated DNA with either a mixture of two ‘homemade' REST antibodies (REST14 and REST17, see above) or a commercial REST Ab (Millipore 17-641). We confirmed the specificity of REST ChIP-seq antibodies with western blot analysis in REST knockdown samples compared to controls (Supplementary Fig. 5a). In samples from HCN cells in iQNP and TAP conditions, ChIP experiments were starting with 200 μg of chromatin and 10 μg of anti-REST or 20 μl of ‘homemade' anti-REST serum following the manufacturer's protocol (Covaris). Briefly, nuclei were resuspended in sonication buffer and sonication was performed for 4 min in Covaris CS220. Protein-A beads were used for collecting sonicated DNA pulled down overnight by REST. After several washes, ChIP DNA was eluted from the beads, purified using Qiagen PCR purification kit (28104), and used downstream for ChIP-seq and ChIP-qPCR. The amount of DNA collected was estimated using Qubit dsDNA HS Assay Kit (Life technologies Q32851). Quantitative ChIP-PCR reactions were performed on Applied Biosystems 7,000 detection system using Biorad iTaq Universal SYBR green supermix (172-5124). Enrichment was calculated relative to 5% input at REST-binding site (RE1 site) and non-RE1 sites 3 kb away from the RE1 site/REST-binding site, using the deltadelta cycle threshold method. Primers used for ChIP-qPCR analysis are available upon request.

### RNA isolation and RNA-seq/qPCR

Total RNA was isolated from tissue using Trizol (Invitrogen 15596018). Reverse transcription was carried out using Biorad iScript cDNA synthesis kit (170-8891). Quantitative PCR reactions were performed on Applied Biosystems 7,000 detection system using Biorad iTaq Universal SYBR green supermix (172-5124). Normalization was based on the expression of beta-actin and relative gene expression or relative mRNA level was determined using the deltadelta cycle threshold method. Primers used for quantitative reverse transcription–PCR analysis are available upon request. For RNA-seq, total RNA was isolated from tissue using miRNAeasy (Qiagen 217004) and on column DNAseI digestion was performed (Qiagen 79254).

### RNA-seq and ChIP-seq data analysis

RNA-seq (*N*=2 each for Ctrl electroporation in iQNP and TAP conditions; *N*=4 each for REST knock-down electroporation in iQNP and TAP conditions) and ChIP-seq (*N*=1 for each, ‘homemade' REST antibodies mix and commercial REST antibody for iQNP and TAP conditions; See Supplementary Table 2) was performed on the Illumina platform. Then we selected peaks common to both runs to increase stringency of our analysis with respect to detection of most consistent peaks. The ChIP libraries were prepared and sequenced on Illumina platform following the manufacturer's protocols. All bioinformatics analysis was performed using default settings unless noted otherwise. Eighty-eight to 91.7% of 33 to 38 million single end ChIP-seq reads were mapped to rat rn4 genome using Bowtie2. Peak discovery and identification of peaks common to both antibodies of REST run separately in ChIP-seq was determined by using HOMER peak calling and merge tools, respectively. For each condition, we selected REST bound sites (peaks) overlapping between multiple REST antibodies using HOMER peak calling and merge peaks analyses^58^ (Supplementary Fig. 5b,c). A smaller number of REST bound sites in iQNP and TAP conditions did not overlap between multiple REST antibodies and was not used in downstream analyses (Supplementary Fig. 5b,c). Motifs were determined by *de novo* motif discovery using HOMER motif tool. Ngs.plot tool was used to plot heat maps of REST binding and REST enrichment was calculated relative to 5% Input for normalized graphical visualization. Using ChIP-seek online tool, peaks were annotated to genes and genes within TSS +/−10 kb were intersected with gene expression profile obtained from RNA-seq. From HCN cells in iQNP and TAP conditions transduced with REST shRNA knockdown vector or control vector, isolated RNA library was prepared and sequenced on Illumina platform following the manufacturer's instructions. Sixty-seven to 71% of 78 to 145 million pair end RNA-seq reads were mapped to rat rn4 genome using Tophat2. Cufflink and cuffdiff was used to estimate Fragments Per Kilobase Of Exon Per Million Fragments Mapped and determine significant differential gene expression. R-studio heatmap.2 was used for heat map representation of RNA-seq data and hierarchical clustering. ChIP-seq peaks annotated to TSS +/−10 kb in REST iQNP and REST TAP conditions were intersected with cuffdiff RNA-seq output (gene expression). iQNP and TAP genes bound by REST whose gene expression was upregulated in REST knockdown RNA-seq relative to control electroporation, were selected to identify unique and common REST-dependent targets in iQNP and TAP. Panther GO classification system with default settings, which generated *P* values after multiple test adjustments was used for GO analysis on unique and common REST targets in iQNP and TAP. Using ngs.plot the input normalized enrichment of binding at these genes was determined and plotted, while UCSC genome browser was used for visualization of RNA-seq and ChIP-seq data of the target genes.

### BrUTP rRNA synthesis assay

BrUTP assay was used to measure rRNA synthesis following a published protocol specifically developed for monolayer cell culture system. Briefly, adherent HCN cells were pretreated for 2 h with 10 μg ml^−1^ alpha-amanitin (an inhibitor of extranucleolar RNA) to assay rRNA synthesis (nucleolar RNA) and with 0.05 μg ml^−1^ ActinomycinD as a negative control. The pretreated adherent cells were lipofected with DOTAP-BrUTP (1.5 mM) complexes prepared in media for 5 min and incubated with adherent cells for 15 min followed by 1 h incubation of the adherent cells in fresh media. Thereafter, cells were washed and fixed in 4% PFA for 20 min at RT and BrUTP staining was performed by general immunohistochemistry protocol as described above and were visualized using a confocal laser-scanning microscope AIR from Nikon equipped with four laser lines (405, 488, 561 and 633 nm) under 40 × objective lens. BrUTP punctuate labelling intensity, number and area occupied were quantified using ImageJ software. A detailed list of antibodies is available in Supplementary Table 2.

### Statistics

All statistical analysis was performed using GraphPad Prism 6. *In vitro* immunocytochemisty and PI-flow cytometry (TAP versus iQNP, TAP versus TAP', and control EGFP versus REST shRNA-EGFP within iQNP and TAP conditions), qPCR (iQNP versus TAP, and control EGFP versus REST shRNA-EGFP within iQNP and TAP conditions) and ChIP-qPCR (non-RE1 iQNP versus TAP conditions, and RE1 iQNP versus TAP conditions) experiments were analysed for statistical significance using an unpaired, two-tailed Student's *t*-test with data expressed as mean ±s.e.m. Results from lentivirus hGFAP-Cre-p2A-mCh injections of REST fl/fl and WT mice at 2 dpi and 7 dpi were analysed for statistical significance using Kruskal–Wallis nonparametric test, which included Dunn's multiple comparison test and generated exact *P* values. Results from candidate genes overexpression, lentivirus hGFAP-cDNA-IRES-mCh and control lentivirus hGFAP-IRES-mCh injections of WT mice at 7 dpi were analysed for statistical significance using Kruskal–Wallis nonparametric test, which included Dunn's multiple comparison test and generated exact *P* values. *P* values *P≤0.05 were considered significant.

Statistical analysis for ChIP-seq data to detect REST peaks and motifs in iQNP and TAP conditions was done by HOMER, with significant peaks selected that had a Poisson *P* value ≤1.00e-04 and significant motifs that had a *P* value≤1.00e-11. Statistical analysis of differential gene expression between REST shRNA knockdown and control transduced cells iQNP and TAP conditions was determined by using Cufflink/Cuffdiff and fold changes≥2 with *P* value≤0.02 and *q*-value≤0.05 were considered significant.

### Data availability

Gene Expression Omnibus (GEO) database series accession codes for data sets generated and used in this study are GSE 70695 (ChIP-seq) and GSE 70696 (RNA-seq). The rest of the data supporting the conclusions of this data are available from the corresponding author upon request.

## Additional information

**How to cite this article:** Mukherjee, S. *et al*. REST regulation of gene networks in adult neural stem cells. *Nat. Commun.* **7,** 13360 doi: 10.1038/ncomms13360 (2016).

**Publisher's note:** Springer Nature remains neutral with regard to jurisdictional claims in published maps and institutional affiliations.

## Supplementary Material

Supplementary Information Supplementary Figures 1-8 and Supplementary Tables 1-2.

Supplementary Data 1 Antibody List

Supplementary Data 2 Cuffdiff Control and Rest shRNA knock-down (kd) iQNP conditions (electroporation) and differentially expressed genes atleast 2-fold in iQNP Rest kd relative to iQNP Control or atleast 2-fold in iQNP Control relative to iQNP Rest kd.

Supplementary Data 3 Cuffdiff Control and Rest shRNA knock-down (kd) TAP conditions (electroporation) and differentially expressed genes atleast 2-fold in TAP Rest kd relative to TAP Control or atleast 2-fold in TAP Control relative to TAP Rest kd.

Supplementary Data 4 ChIP-seq Analysis of Rest HOMER peak calling in iQNP conditions.

Supplementary Data 5 ChIP-seq Analysis of Rest HOMER peak calling in TAP conditions.

Supplementary Data 6 De novo Motif Analysis of Rest Peaks in iQNP conditions.

Supplementary Data 7 De novo Motif Analysis of Rest Peaks in TAP conditions.

Supplementary Data 8 ChIP-seq Analysis of Rest and Genomic Annotation in iQNP conditions.

Supplementary Data 9 ChIP-seq Analysis of Rest and Genomic Annotation in TAP conditions.

Supplementary Data 10 ChIP-seq Rest iQNP an TAP, Genomic Annotation +/-10kb TSS and corresponding RNA-seq values atleast 2-fold UPREGULATED in Rest knockdown relative to control electroporation in iQNP and TAP conditions.

Supplementary Data 11 GO analysis Unique iQNP Targets.

Supplementary Data 12 GO analysis Unique TAP Targets.
