# Supplementary material for: REST regulation of gene networks in adult neural stem cells
Source: Nat Commun. 2016 Nov 7;7:13360. doi: 10.1038/ncomms13360 (PMC5103073; doi:10.1038/ncomms13360)
Supplement: Supplementary Information — Supplementary Figures 1-8 and Supplementary Tables 1-2. [file ncomms13360-s1.pdf]

SUPPLEMENTARY FIGURES

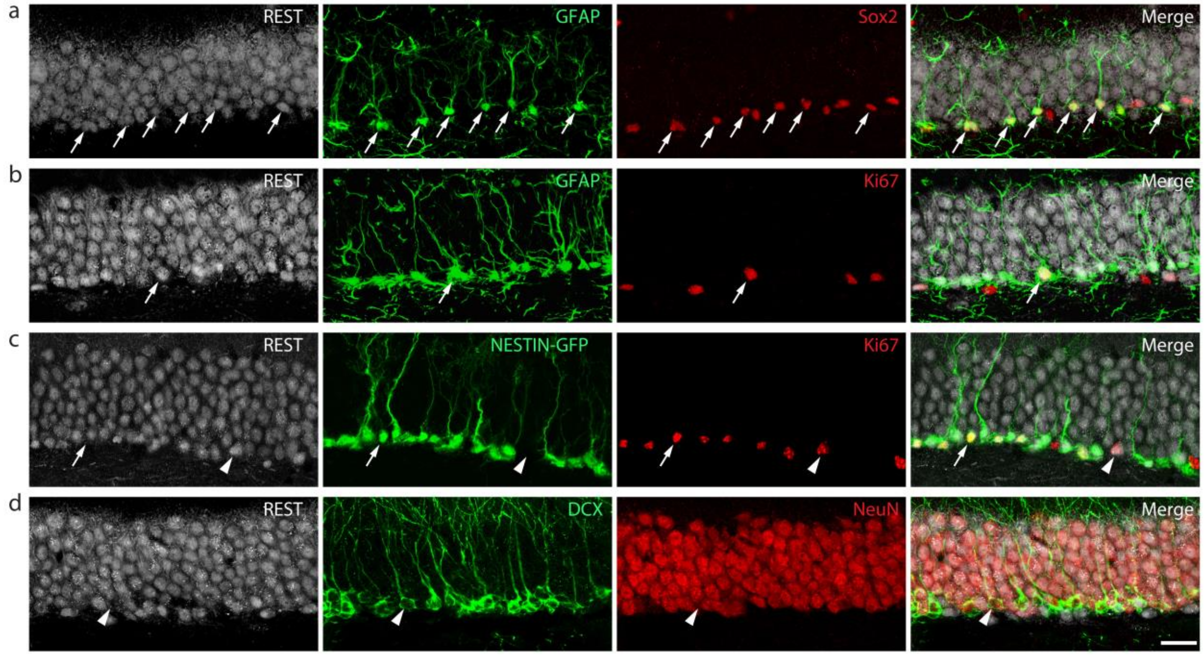

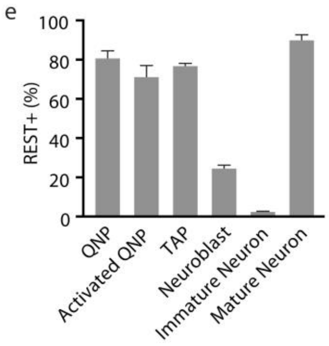

f

	Morphology	Antibody	
QNP	radial process	GFAP+Sox2+	na
Activated QNP	radial process	GFAP+Ki67+	na
TAP	no radial process, round soma	Ki67+	NestinGFP+
Neuroblasts	na	Ki67+	NestinGFP-
Immature Neuron	na	DCX+	na
Mature Neuron	na	NeuN+	na

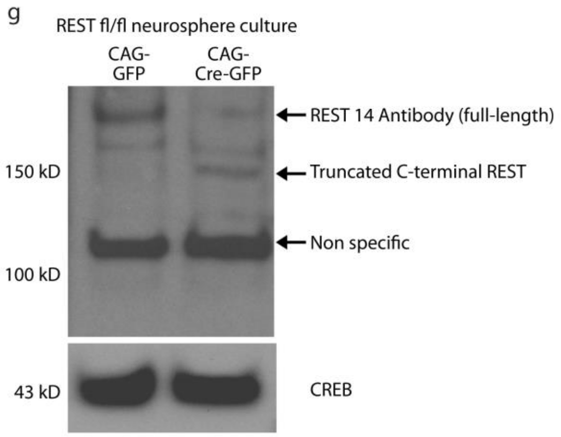

Supplementary Figure 1, Adult hippocampal QNPs and TAPs uniformly express REST

a-b) Confocal images of adult hippocampal mouse sections showing GFAP (green), Sox2 (red), and REST (grey) triple-labeled QNP cells (arrows) (a) or GFAP (green), Ki67 (red), and REST (grey) triple-labeled activated QNP cells (arrows) (b). c) A representative section of an adult Nestin-GFP+ hippocampus. Arrow indicates a Nestin-GFP without radial process (green), Ki67 (red), and REST (grey) triple-labeled TAP cell. Arrowhead indicates a Nestin-GFP-, Ki67 (red), and REST (grey) triple-labeled neuroblast. d) REST (grey) staining was detected in NeuN (red) granule neurons but was almost absent from DCX (green) immature neurons. e) Quantification as percentage of REST co-labeled cells out of total QNPs, activated QNPs, TAPs, neuroblasts, immature neurons, and mature neurons. f) Table of morphology criteria and antibody combination of markers or transgenic reporter mice used to identify subtypes of neural stem cells and granule neurons in adult hippocampus. g) Western blot result from *in vitro* REST fl/fl neurospheres infected with Adenovirus CAG-GFP or CAG-Cre-GFP showing full length REST and truncated C-terminal REST peptide, respectively. For all quantifications, data are plotted as the mean ± SEM (*$p \leq 0.05$ and ns = not significant). Scale bar in d: 20μm. One representative image from three independent WT control and Nestin-GFP transgenic mice are shown. NA = not applicable.

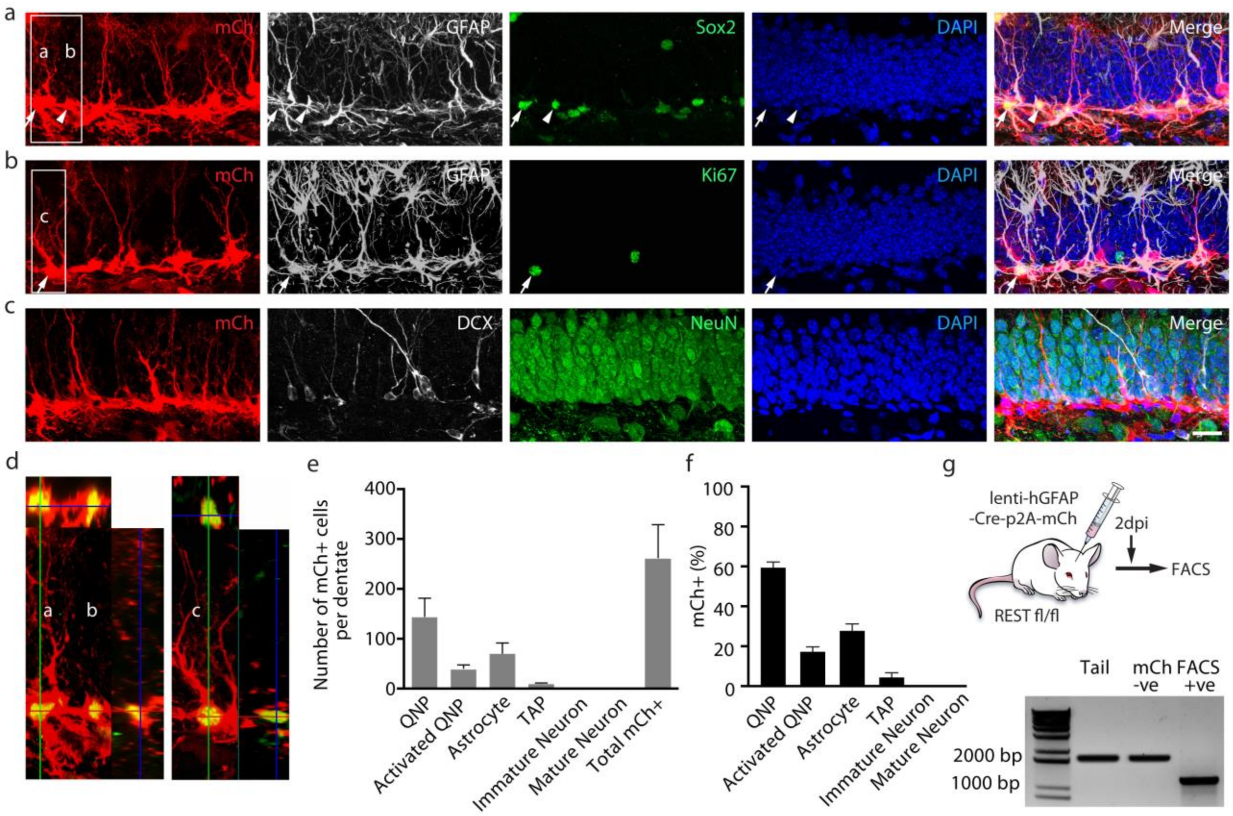

Supplementary Figure 2, Related to Figure 1: Specificity of lentivirus hGFAP-Cre-mCherry and confirmation of REST knockout efficiency

Lentivirus hGFAP-Cre-p2A-mCh injections were performed in hippocampal dentate gyrus of 6-8-week old WT mice. The resulting infected cells at 2dpi are shown. a-b) Confocal images of adult hippocampal mouse sections showing mCh (red), GFAP (grey), and DAPI (blue) labeled cells co-expressing a) Sox2 (green) or b) Ki67 (green). Quadruple-labeled a) QNP cells (arrow) or astrocyte cell (arrowhead) (inset d) or b) activated QNP cells (arrow). b) mCh (red) staining was almost absent from GFAP-Ki67+ (green) TAP cells. c) mCh (red) staining was almost absent from DCX+ (grey) cells and NeuN+ (green) granule neurons. e) Quantification of the total number of mCh+ QNP cells (inset a: mCh (red), Sox2 (green)), activated QNP cells (inset c: mCh (red), Ki67 (green)), astrocyte cells (inset b: mCh (red), Sox2 (green)), TAP cells, immature neurons and mature neurons per dentate gyrus. f) Quantification of the percentage of

mCh+ co-labeled cells out of the total mCh+ cells. g) Genotyping PCR result from FACS sorted REST fl/fl tail, mCh-ve and mCh+ve cells to show *in vivo* deletion of REST by lenti-hGFAP-Cre-p2A-mCh resulting in 1.2 kb band for the targeted allele and 2.8 kb band for the intact REST fl/fl allele. For all quantifications, data are plotted as the mean ± SEM (*p ≤ 0.05 and ns = not significant). Scale bar in c: 20μm. One representative image from three independent WT genotype mice is shown.

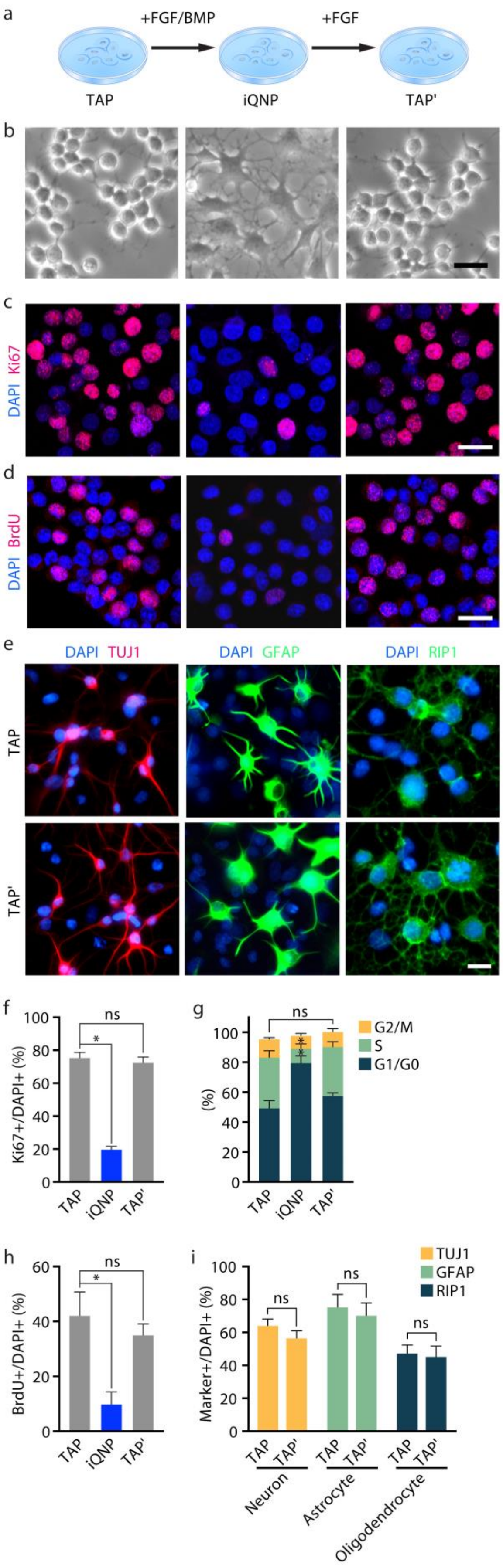

Supplementary Figure 3, *In vitro* model of quiescence

a) HCN cells treated with FGF-2 and BMP4 become quiescent (iQNP). HCN cells can be maintained in a proliferative state (TAP) or iQNPs can be reactivated (TAP') with FGF-2 treatment. b) Brightfield images of TAP, iQNP and TAP' HCN cells. c-d) Immunofluorescent analysis of TAP, iQNP and TAP' HCN cells fixed at 3 days (iQNP and TAP) or 6 days (TAP' only) *in vitro* and stained with proliferation markers, c) Ki67 (red) and d) BrdU (red). e) Immunofluorescent analysis of TAP and TAP' HCN cells fixed after growing in differentiation conditions *in vitro* and stained with differentiation markers, Tuj1 (neuronal marker, red), GFAP (astrocyte marker, green) and RIP1 (oligodendrocyte marker, green). Quantification of f) Ki67 and h) BrdU as percent of all cells (DAPI+). g) Cell cycle analysis by PI flow cytometry of TAP, iQNP and TAP' HCN cells. i) Quantification of Tuj1, GFAP and RIP1 as percent of all cells (DAPI+). For all

quantifications, data are plotted as the mean ± SEM ($^*p \leq 0.05$ and ns = not significant). Scale bars in b, c, d and e: 20μm. All experiments were performed at least three times independently. Experiments were analyzed for statistical significance using an unpaired, two-tailed Student's t-test.

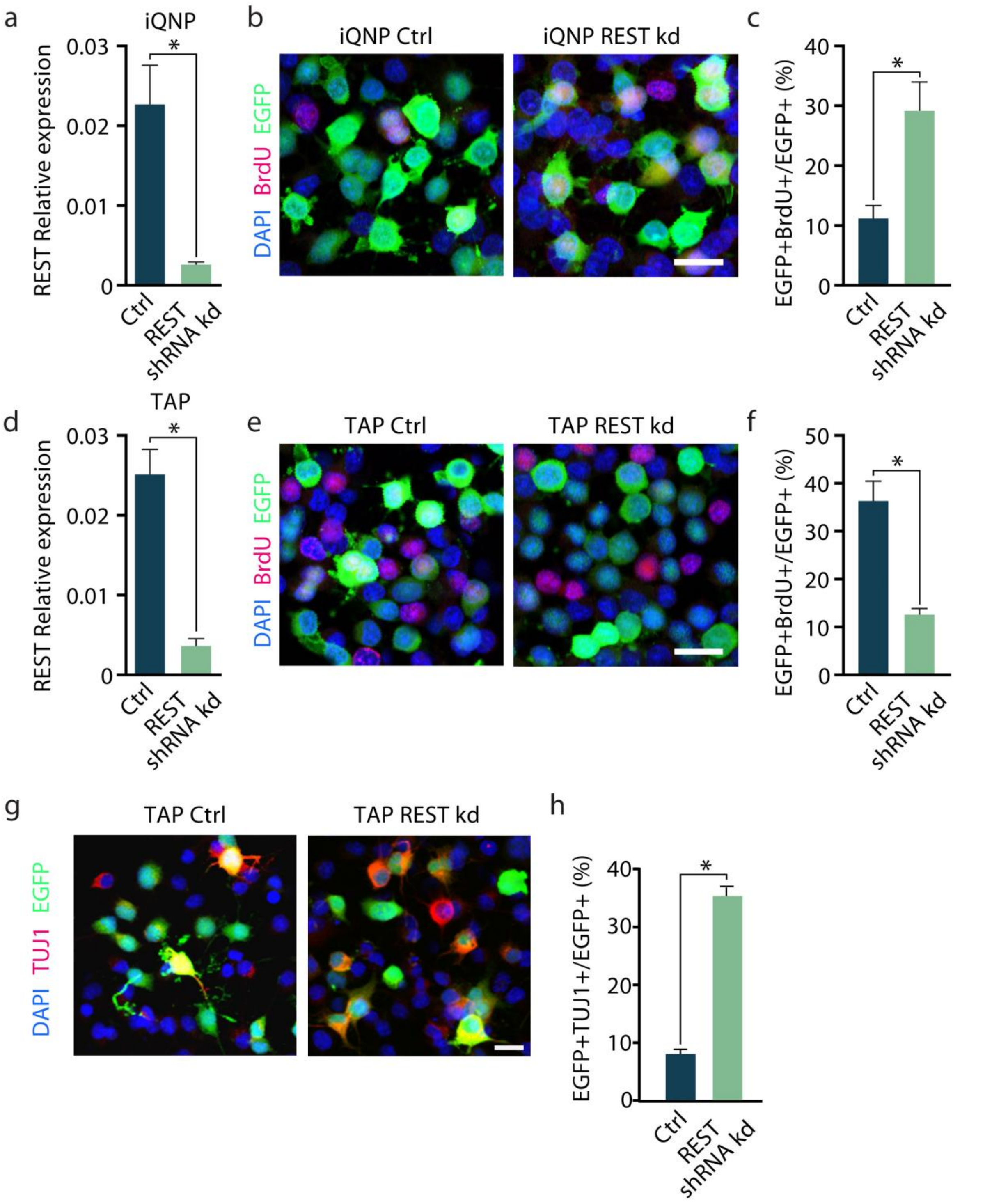

Supplementary Figure 4, Related to Figure 3: REST is required for maintenance of iQNP and TAPs *in vitro*

To confirm efficiency of REST knockdown *in vitro*, HCN cells in iQNP and TAP conditions were electroporated with a control EGFP or REST shRNA-EGFP vector. a,d) qPCR validation of REST mRNA knockdown in REST shRNA electroporated compared to control electroporated cells in iQNPs (a) and TAPs (d). Immunofluorescent analysis and quantification of electroporated cells fixed at 2.5 days *in vitro* and stained with proliferation markers, b) BrdU (red) in iQNP condition, e) BrdU (red) in TAP condition or neuronal differentiation marker g) Tuj1 in TAP condition. Quantification of electroporated c) BrdU in iQNP conditions, f) BrdU in TAP conditions and h) Tuj1 in TAP condition as percent of all electroporated cells (EGFP+). For all quantifications, data are plotted as the mean ± SEM (*$p \leq 0.05$ and ns = not significant). Scale bars in b, e and g: 20μm. All experiments were performed at least three times independently. Experiments were analyzed for statistical significance using an unpaired, two-tailed Student's t-test.

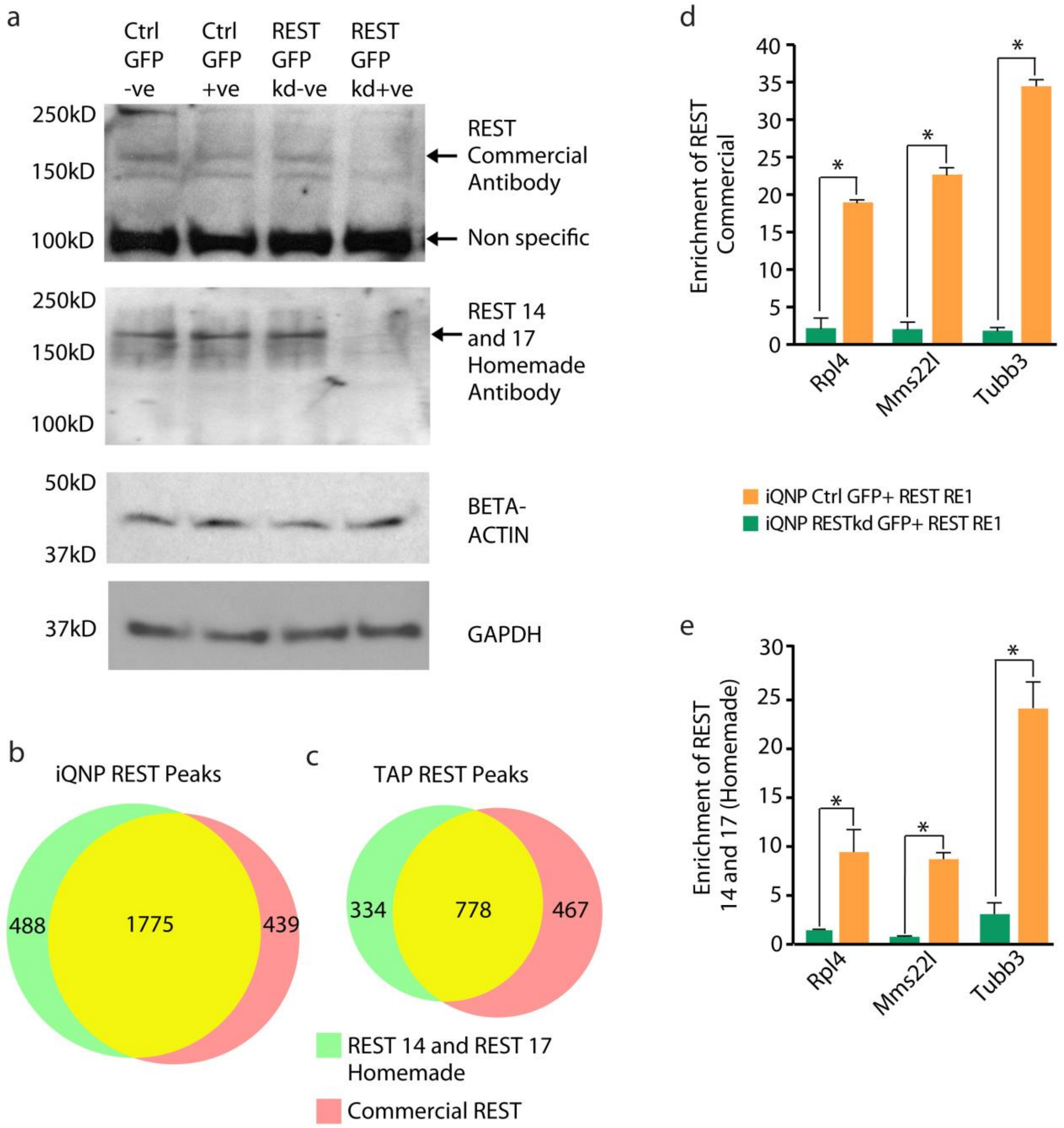

Supplementary Figure 5, Related to Figure 5: Validation of REST antibodies used in ChIP-seq studies

a) Western blot result from *in vitro* REST shRNA and control vector transduced and FACS sorted iQNP HCN cells probed with the commercial REST antibody showing full length REST and non-specific band or probed with REST14 and 17 antibodies. Beta-actin and GAPDH served

as loading controls. b-d) Venn-diagram overlap of REST bound peaks obtained with either REST14 and 17 antibodies or the REST commercial antibody in iQNP ChIP-seq (b) and TAP ChIP-seq (c). d,e) ChIP-qPCR validation of select REST target genes using the commercial REST (d) or REST14 and 17 (e) antibodies from *in vitro* REST shRNA and control vector transduced and FACS sorted iQNP HCN cells. For all quantifications, data are plotted as the mean ± SEM (*$p \leq 0.05$ and ns = not significant). All experiments were performed at least two times independently. Experiments were analyzed for statistical significance using an unpaired, two-tailed Student's t-test.

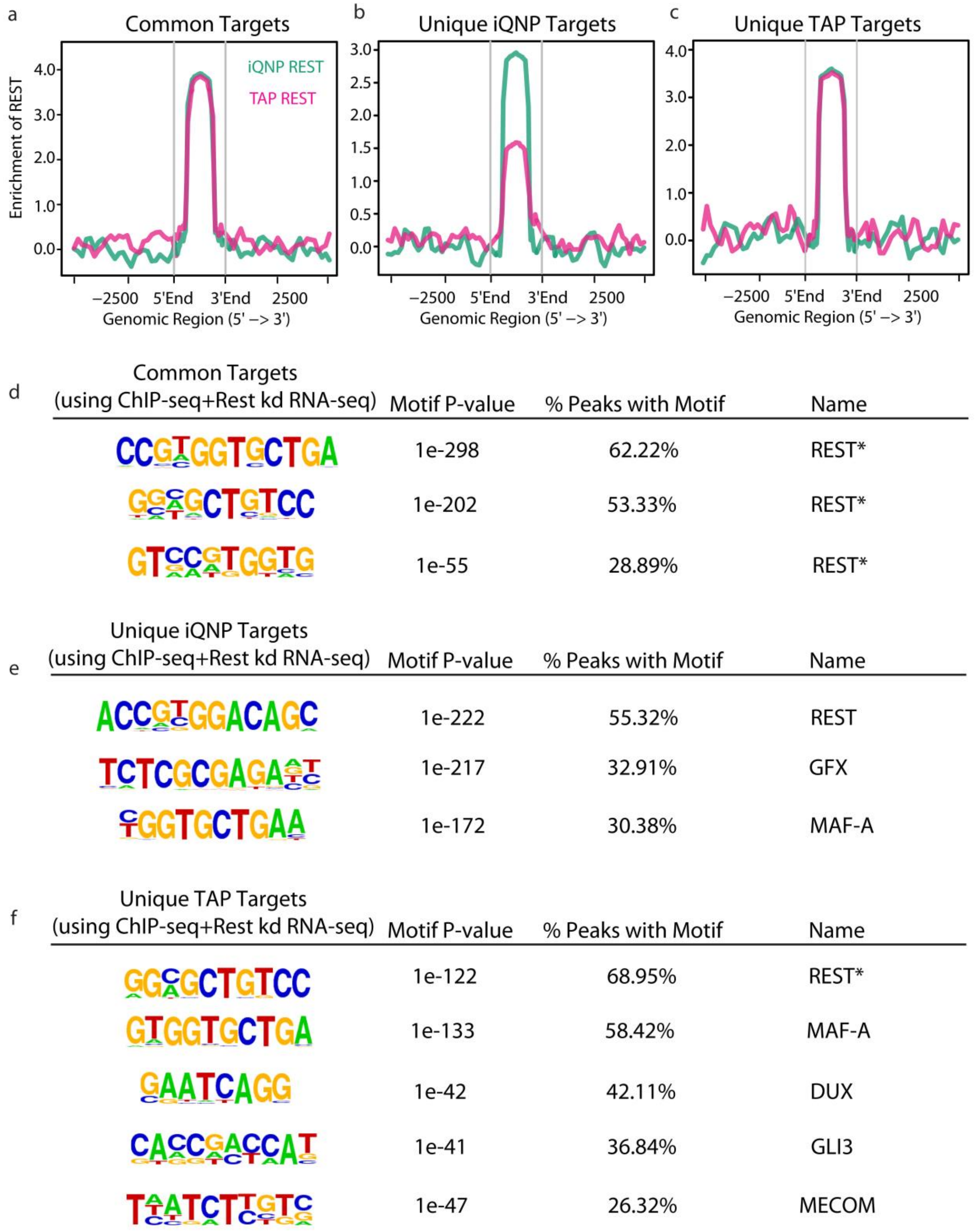

Supplementary Figure 6, Related to Figure 5: REST binding sites on unique and common iQNP and TAP targets

a-c) Enrichment of REST (normalized to 5% Input) at a) common targets, b) unique iQNP targets and c) unique TAP targets. d-f) De novo identified motifs by HOMER in common targets (d), unique iQNP targets (e) and unique TAP targets (f).

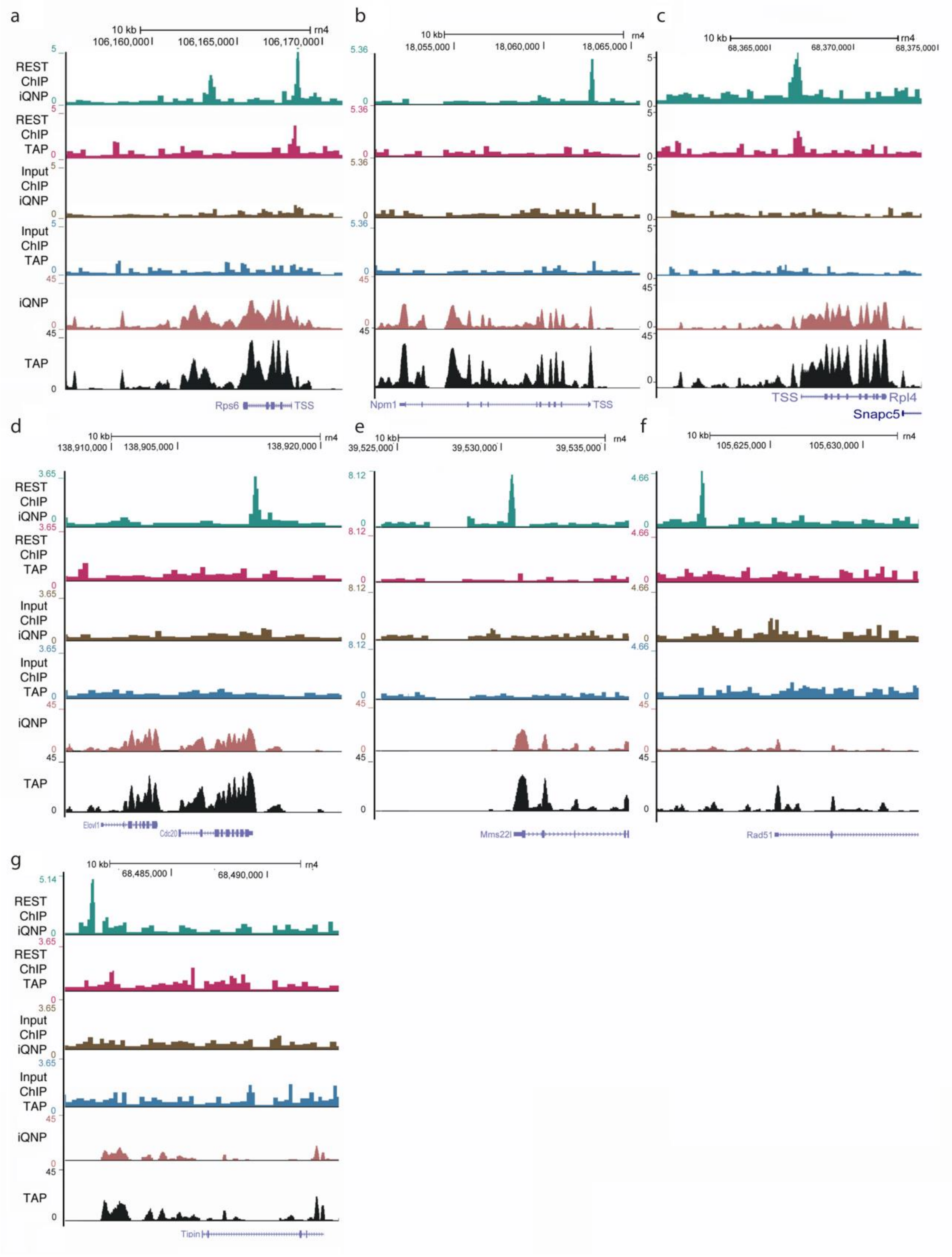
a
b
c
d
e
f
g
REST ChIP iQNP
REST ChIP TAP
Input ChIP iQNP
Input ChIP TAP
iQNP
TAP
10 kb
rn4
Rps6
TSS
Npm1
Rpl4
Snapc5
Elovl1
Cdc20
Mms22l
Rad51
Tipin

Supplementary Figure 7, Related to Figure 5: REST represses ribosome biogenesis and cell cycle genes in iQNP conditions

a-g) UCSC genome browser snapshot of REST target ribosome biogenesis and cell cycle genes: Rps6 (a), Npm1 (b), Rpl4 (c), Cdc20 (d), Mms22l (e), Rad51 (f), and Tipin (g) loci showing REST ChIP-seq and RNA-seq results.

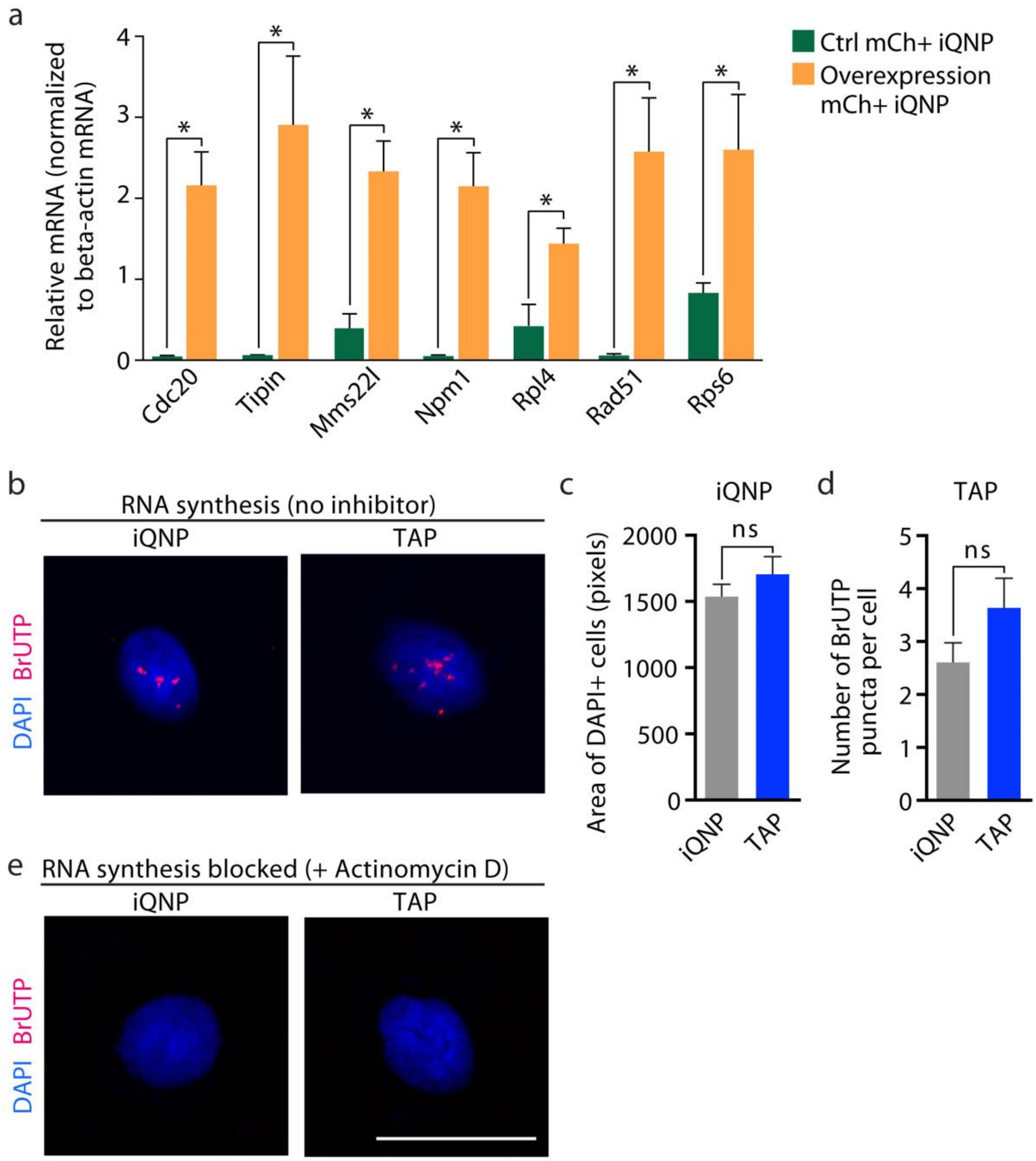

Supplementary Figure 8, Related to Figure 6: Confirmation of REST target gene overexpression and control BrUTP experiments.

a) qPCR validation of overexpression of Cdc20, Tipin, Mms22l, Rad51, Npm1, Rpl4 and Rps6 mRNA in iQNP conditions electroporated with a control lenti-hGFAP-IRES-mCh or overexpression lenti-hGFAP-cDNA-IRES-mCh vector. b,e) Immunofluorescent analysis of

BrUTP+(red)DAPI+(blue) in iQNP and TAP conditions without any inhibitor (b) or in presence of actinomycin D inhibitor (e). c,d) Quantification of area occupied by the whole cell DAPI+ (c) and number of labeled BrUTP puncta (d) in iQNP and TAP conditions. For all quantifications, data are plotted as the mean ± SEM (*p ≤ 0.05 and ns = not significant). Scale bar in e: 20μm. All experiments were performed at least three times independently. ns = not significant. Experiments were analyzed for statistical significance using an unpaired, two-tailed Student's t-test.

SUPPLEMENTARY TABLE

GO term	p-value	gene symbol
**Unique QNP targets**		
1) Cation transport/ion transport	9.91E-05	Gabrg1, Gpr158, Hcn3, Kcnk3, Kcnk12, Kcnh4, Scn3b, Slc39a7, Svop
2) rRNA metabolic process/ ribosome biogenesis	2.91E-03	Npm1, Rps7, Rps6, Rpl10a, Rpl4
3) Translation	1.33E-02	Ddx25, Rpl26, Rps6, Rps7, Rps19, Rpl27a, Rpl10a, Rpl4
4) Response to stimulus	2.94E-02	Cxcl11, Gpr149, Hrh3
5) Cell-cell signaling	1.14E-01	Gabrg1, Gpr149, Hrh3, Syp
6) Cell cycle	1.50E-01	Actl6b, Cabp1, Cdc20, Cdk5r2, Dctn2, Mms22l, Qtrt1, Rad51, Tipin
**Unique TAP targets**		
1) Ion transport	1.18E-04	Atp1a3, Cacna1b, Gria2, Kcnq2, Kcnc1
2) Cation transport	6.63E-04	Atp1a3, Cacna1b, Kcnq2, Kcnc1
3) Homeostatic process/ cell-cell adhesion	8.78E-03	Atp1a3, Col5a3
4) Regulation biological process	4.01E-02	Cacna1b, Gria2, Gsx1, Kcnc1, Kcnq2
**Common targets**		
1) Synaptic transmission	2.75E-03	Chrnb2, Cplx1, Diras1, Snap25
2) Ion transport	1.04E-02	Chrnb2, Grin1, Hcn2, Kcnh6, Slc39a3
3) Exocytosis	1.09E-02	Disp2, Snap25, Sncb
4) Cation transport	2.20E-02	Chrnb2, Hcn2, Kcnh6, Slc39a3
5) Neurological system process	2.43E-02	Celf6, Chrnb2, Cplx1, Diras1, Hpca, Snap25
6) Intracellular protein transport	4.03E-02	Disp2, Diras1, Rgs20, Sncb, Tubb3
7) Nervous system development	5.39E-02	Celsr3, Celf6, Chrnb2, Stmn3

Supplementary Table 1: Gene Ontology showing candidate REST targets upregulated in REST knockdown only in iQNPs (unique iQNP targets), only in TAPs (unique TAP targets) and in both (common targets). Highlighted genes indicate selected iQNP targets for subsequent validation and functional studies.

Antibody Name	Company (Catalogue Number)	Dilution	Technique used
Anti-REST, REST14	Inhouse, Cocalico Biologicals, Inc.	1:500	IHC, WB
Anti-REST, two antibodies REST14, REST17 mixed 1:1 for all uses	Inhouse, Cocalico Biologicals, Inc.	1:40 of each	ChIP
Anti-REST commercial	Millipore (17-641)	10ug	ChIP
Anti-GFAP	Millipore (MAB360)	1:500	IHC
Anti-Sox2	Santa cruz (sc-17320)	1:500	IHC
Anti-Ki67	Neomarkers (RM-9106)	1:250	IHC, ICC
Anti-DCX	Millipore (AB2253)	1:500	IHC
Anti-NeuN	Chemicon (MAB377)	1:1000	IHC
Anti-mCh	Novus (NBP1-96752)	1:500	IHC, ICC
Anti-mCh	Life Technologies (M11217)	1:500	IHC, ICC
Anti-DsRed (also detect mCh)	Clontech (632496)	1:500	IHC, ICC
Anti-GFP	Aveslabs (GFP-1020)	1:1000	IHC, ICC
Anti-IdU	Sigma (SAB3701448)	1:250	ICC
Anti-BrdU	Accurate (OBT0030)	1:250	ICC
Anti-TUJ1	Covance (E10344JF)	1:7500	ICC
Anti-RIP1	Hybridoma Bank	1:500	ICC
Anti-RIP2	Hybridoma Bank	1:500	ICC
Anti-CREB	Cell Signaling (9197)	1:1000	WB

Supplementary Table 2: Antibody List. Abbreviations used, IHC=Immunohistochemistry, ICC=Immunocytochemistry, WB=Western Blot, ChIP=Chromatin immunoprecipitation.
